# T_1_, T_2_, and complex permittivities of hydrogels, paramagnetic salt solutions, and oils at 0.35, 1.5, and 3 Tesla

**Published:** 2026-08-11

**Authors:** H. Michael Gach, Kerri Prinos, Hannah Sechaga

**Affiliations:** Departments of Radiation Oncology, Radiology, and Biomedical Engineering, Washington University in St. Louis, St. Louis, Missouri 63110; Department of Electrical and Systems Engineering, Washington University in St. Louis, St. Louis, Missouri 63110; Department of Radiation Oncology, Washington University School of Medicine, St. Louis, Missouri 63110

**Keywords:** Relaxation time, dielectric strength, electrical conductivity, MRI, phantom liquids

## Abstract

**Approach:**

The T_1_, T_2_, and complex permittivities of candidate MRI phantom constituents (hydrogels, paramagnetic electrolytes, and oils) were measured at different magnetic field strengths and concentrations.

**Data:**

T_1_ decreased with increasing concentration except for PEG. The T_1_ and T_2_ relaxivities of polyethylene glycol (PEG), polyvinylpyrrolidone (PVP), sodium polyacrylate, and deionized water did not significantly vary with field strength. The T_2_ relaxivities of gelatin and sodium alginate did not significantly vary with field strength. All of the hydrogels and paramagnetic electrolyte solutions had high static dielectrics (e.g., ε_s_~70) similar to water. The static electrical conductivities of Miller's LB agar, gelatin, PVA, sodium alginate, sodium polyacrylate, xanthan gum, CuSO_4_, NiCl_2_, and Mn(NO_3_)_2_ rose with concentration. However, the conductivities of PEG and PVP did not rise with concentration.

**Conclusions:**

Sodium polyacrylate, PVP, and PEG hydrogels are challenging for generating consistent phantoms. Sodium alginate, Miller's LB agar, and xanthan gum were good hydrogel candidates. Mn(NO_3_)_2_ had the highest relaxivities of the tested samples. All three oils (canola, castor, and grapeseed) are good candidates for low dielectric (ε_s_<10) phantoms for high field applications) but with low conductivities.

## 1. INTRODUCTION

Phantoms are an essential tool for MRI quality assurance (QA). A large variety of commercial MRI phantoms are available. However, commercial phantoms can be expensive especially for MRI facilities with limited funding. Home-built phantoms may be satisfactory for many applications and their materials may be cheap.

The design of a phantom requires knowledge about the material relaxation times and permittivities. Phantom materials include water-based liquids doped with paramagnetic electrolytes, oils, and gels. Material properties like relaxation times and permittivities are often hard to find at clinical Larmor frequencies.

The purpose of this study was to measure and compare the T_1_, T_2_, and complex permittivities of hydrogels, paramagnetic electrolytes, and oils commonly used in MRI phantoms. Many of the relaxation publications occurred prior to the era of 1.5 and 3 T clinical MRI. Our measurements cover nominal clinical field strengths (1.5 and 3 T) and 0.35 T used in MRI-guided radiation therapy (MRgRT). Other desirable clinical field strengths include 7 T (high field) and 0.55 T (low field). Therefore, extrapolations (7 T) and interpolations (0.55 T) of R_1_ and r_1_ were performed and are included below.

## 2. METHODS

Relaxation times and relaxivities were measured at 21°C for three field strengths: 0.35 T (ViewRay MRIdian MRI-Linac, VB19), 1.5 T (Philips Ingenia MRI, V5.7), and 3 T (Siemens mMR PET-MRI, VE11P).

T_1_ was measured using an inversion recovery (IR) echo planar imaging (EPI) sequence with 26–30 variable inversion times (TIs) ranging from 21 ms to 5 s. T_2_ was measured using a Carr–Purcell–Meiboom–Gill (CPMG) multiple spin-echo sequence with 32 echoes and echo spacing (ΔTE) of 20 ms except for deionized water (ΔTE = 40 ms) and Mn(NO_3_)_2_ samples (ΔTE = 12.2, 9 and 8.8 ms for 0.35, 1.5, and 3 T, respectively). The pulse sequence parameters are listed in Tables 1 and 2. A single slice was acquired to minimize the effects of crosstalk and off-resonance saturation on the results.

T_1_ and T_2_ values were fit to their respective three parameter models using a nonlinear fit and Mathematica (Wolfram, V11.1):^1^

(1) $$S(TI)=A\cdot e^{-\frac{TI}{T_{1}}}+B$$

(2) $$S_{corr}(TE)=C\cdot \left[1-2e^{\frac{-\left(TR-\frac{TE}{2}\right)}{T_{1fi}}}+e^{\frac{-TR}{T_{1fit}}}\right]e^{-\frac{TE}{T_{2}}}+D$$

where TR is the repetition time, TE is the echo time, TI is the inversion time, and *A, B, C, D* are fit variables. $T_{1fit}$ resulted from solving Eq. (1).

T_1_ values were interpolated (0.55 T) and extrapolated (7 T) T_1_ from the acquired data using:^2^

(3) $$T_{1}\left(B_{0}\right)=A\cdot {B_{0}}^{C}$$

where *A* and *C* are fit variables and $B_{0}$ is the field strength. The interpolated and extrapolated T_1_ values were converted into relaxation rates (R_1_) and relaxivities (r_1_).

### Hydrogels:

Hydrogel concentrations were based on prior study ranges, preparation constraints, and our ability to acquire relaxation and permittivity data from the samples. Hydrogels included sodium polyacrylate (Educational Innovations, Water-Loc^™^, Item No. GB-6A, $19.95/454 g, Supplier: TeacherSource, Concentrations: 0.25–5% w/w)^3^, xanthan gum (MP Biomedicals, Stock No. ICN960002180, CAS 11138–66-2, $76.80/100 g, molecular weight (MW): >2,000,000 g/mol, Supplier: Fisher Scientific, Concentrations: 0.5–5% w/w)^4^, polyvinyl alcohol (PVA, Aldon Corp SE, Stock No. 470302–040, CAS 9002–89-5, $40.15/500 g, MW: 100,000 g/mol, Supplier: VWR, Concentrations: 0.5–5% w/w)^5^, polyethylene glycol (PEG) 8000 (ThermoFisher, Stock No. 043443.36, CAS: 25322–68-3, $58.20/500 g, molecular weight (MW): 62.07 g/mol, Supplier: Fisher Scientific, Concentrations: 1–20 mM)^6^, and polyvinylpyrrolidone (PVP, K30 (VWR, Stock No. 97061–820, CAS 9003–39-8, $103.13/500 g, MW: 40,000 g/mol, Supplier: VWR, Concentrations: 10–30% w/w).^7^ The gelling agent was combined with deionized water at room temperature and stirred vigorously for a period of time that resulted in homogeneity (i.e., 10 to 180 minutes).^5,8,9^ Deionized water (14.3 MΩ-cm) was acquired from a Purelab Pulse deionizer (Elga Labwater, UK).

Hydrogels were also prepared by mixing granulated Miller's LB agar (Fisher BioReagents, Lot No. 145037, $223/500 g, Supplier: Fisher Scientific, Concentrations: 0.5–5% w/w)^10^ or gelatin powder (Aldon Corp SE, Stock No. 470301–134, CAS 9000–70-8, $35.25/500 g, Supplier: VWR, Concentrations: 0.5–5% w/w)^11^ with deionized water at room temperature. Miller's LB agar is a nutrient-rich microbial growth medium which is 32% w/w agar and 27% w/w NaCl. The mixtures were then heated in a microwave oven until they began to boil.^12,13^ This sequence ensured even distribution of the gelatin powder, in particular. The mixture was then stirred and placed in the freezer at −20°C for 20 minutes to allow the gel to set.

### Paramagnetic electrolytes:

The candidate liquids were aqueous solutions of Mn(NO_3_) _2_ * 4H_2_O, 98% (Alfa Aesar, Stock No. AAA1852130, CAS 20694–39-7, $53.90/250 g, molecular weight (MW): 251.006 g/mol, 98% purity, Supplier: Fisher Scientific), NiCl_2_ * 6H_2_O, 99.3% (metals basis) (Alfa Aesar, Stock No. AA1468722, CAS 7791–20-0, $114.82/100g, MW: 237.71 g/mol, melting point: 140°C- H_2_O, Supplier: Fisher Scientific), and CuSO_4_ * 5H_2_O (Zep Root Killer, CAS 7758–99-8, MW: 249.68 g/mol, $13.55/907 g).^4,14,15^ Concentrations ranged from 0.5 mM to 20 mM for NiCl_2_ * 6H_2_O and CuSO_4_ * 5H_2_O, and 0.01 to 5 mM for Mn(NO_3_) _2_.

### Oils:

The oils tested were food-grade canola oil (365 by Whole Foods Market, Organic Expeller Pressed Canola Oil, Lot No. 1160443, $3.99/473 mL, Supplier: Whole Foods Market, molecular weight (MW): 891.45 g/mol, density 0.915 g/cm^3^, C_57_H_110_O_6_), food-grade grapeseed oil, (Napa Valley Naturals, Grapeseed Oil, Lot No. S4913 1212, $12.29/750 mL, Supplier: Whole Foods Market, MW: 280.445 g/mol, density 0.90 g/cm^3^, C_18_H_32_O_2_), castor oil (Thermo Scientific, Stock No. AAL04224AK, CAS 8001–79-4, $28.45/250 mL, Supplier: Thermo Fisher Scientific, MW: 933.45 g/mol, density 0.961 g/cm^3^, C_57_H_104_O_9_).^16–19^

### Permittivities:

Complex permittivity measurements were performed on a benchtop using an Agilent 85070E high-temperature coaxial dielectric probe (Santa Clara, CA) connected to an Agilent E4991A impedance analyzer with a rated range of 10 MHz to 3 GHz.^20^ Measurements ranged from 14 MHz to 1 GHz in 2 MHz intervals. A four-point calibration (short, open circuit, 50 ohm load, and low-loss capacitor) of the impedance analyzer was performed. A three-point calibration (air, short, and 21.6^0^C deionized water) of the dielectric probe was performed after the impedance analyzer was calibrated. The average temperature of the liquids during the permittivity measurements was measured using an Omega Model FOH201 or SAII fiber optic temperature sensor.

Measurements of the hydrogels (Miller's LB agar, gelatin, xanthan gum, sodium polyacrylate, PVA, and PVP) and MnNO_3_ samples were conducted using the Agilent 85070E probe with an Agilent E5061A network analyzer. A three-point calibration (air, short, and 21.6^0^C deionized water) of the dielectric probe was performed before measurements. Measurements ranged from 14 MHz to 1.5 GHz in 15 MHz intervals. We extrapolated the static dielectrics $\left(\varepsilon_{s}\right)$ and electric conductivities $\left(\sigma_{s}\right)$ down to 14 MHz since the dielectric probe is rated for a range from 200 MHz to 20 GHz with the network analyzer.

The complex permittivity data were fit to the Cole-Cole relaxation model using Mathematica to derive the static dielectric constants $\left(\varepsilon_{s}\right)$ and static electrical conductivities $\left(\sigma_{s}\right)$ .^21–24^ The static electrical conductivity $\sigma_{s}$ was calculated from the imaginary component of the complex permittivity $\text{(}\varepsilon^{\prime \prime }\text{)}$ using:

(4) $$\sigma (\omega )=\varepsilon_{0}\omega \varepsilon^{\prime \prime }(\omega )+\sigma_{s}$$

where $\varepsilon_{0}$ is the permittivity of free space.

Linear fits of $\varepsilon_{s}$ and $\sigma_{s}$ versus concentration [C] were generated as:

(5) $$\varepsilon_{s}\approx \varepsilon_{s,0}+a[C]$$

(6) $$\sigma_{s}\approx \sigma_{s,0}+b[C]$$

where *a* and *b* were the respective linear slopes, and $\varepsilon_{s,0}$ and $\sigma_{s,0}$ were the intercepts.

Linear fits of relaxivities were also calculated for the mixtures based on their concentrations [C] and relaxation rates:

(7) $$R_{n}=\frac{1}{T_{n}}=R_{n,0}+r_{n}[C]$$

where n=1 for T_1_ and n=2 for T_2_, respectively. R_n_ is the relaxation rate, R_n,0_ is the intercept, and r_n_ is the relaxivity. Additional relaxivity terms can be added to Equation 7 to address the combination of additional ingredients.^25^

The DC electrical conductivity $\sigma_{DC}$ for each concentration was measured using an Oakton CON 110 handheld conductivity/TDS/°C/°F meter with RS232. A manual single-point calibration was performed using a potassium chloride 84 μS conductivity standard (LaMotte, Lot No. 1286217, CAS 7447–40-7, $8.95/30 mL, Supplier: Extech Instruments). The average temperature of the liquids during the conductivity measurements was 21.5 +/− 0.1 °C and was measured using the Oakton CON 110.

## 3. RESULTS

Data plots of the relaxation rates, relaxivities, static dielectrics, static electrical conductivities, and DC conductivities are provided in Figures 1–23. Error bars represent uncertainties in nonlinear fits for T_1_ and T_2_ relaxation rates (R_1_ and R_2_, respectively) vs. concentration, and linear fits for T_1_ and T_2_ relaxivities (r_1_ or r_2_, respectively) vs. field strength. Error bars represent uncertainties in linear fits of static dielectrics (ε_s_), and static and DC electrical conductivities (σ_s_ and σ_DC,_ respectively) vs. concentration. Paired t-tests were used to compare σ_s_ and σ_DC_. Correlations were calculated between ε_s_, σ_s_, and σ_DC_. Relaxation times (T_1_, T_2_), relaxivities (r_1_, r_2_), relaxation rate intercepts (R_1,0_, R_2,0_), static dielectric (ε_s_), static conductivity (σ_s_), and DC conductivity (σ_0_) and uncertainties are provided in Tables 3–15.

### Hydrogels:

The T_1_ and T_2_ relaxation rates (R_1_ and R_2_, respectively) as a function of Miller's LB agar concentration are shown in Figures 1a and 1b The T_1_ and T_2_ relaxivities (r_1_ and r_2_, respectively) as a function of field strength are shown in Figures 1c and 1d. Miller's LB agar is a mixture of agar (32% w/w), tryptone (27% w/w), NaCl (27% w/w), and yeast (14% w/w) agar. T_1_, T_2_ was consistent with earlier studies when corrected for agar concentration.^26^

The dielectric strengths and electrical conductivities for different concentrations of Miller's LB agar are shown in Figure 2. The relaxation times, relaxivities, and permittivity measurements for Miller's LB agar are summarized in Table 3.

The T_1_ and T_2_ relaxation rates (R_1_ and R_2_, respectively) as a function of gelatin concentration are shown in Figures 3a and 3b The T_1_ and T_2_ relaxivities (r_1_ and r_2_, respectively) as a function of field strength are shown in Figures 3c and 3d.

The dielectric strengths and electrical conductivities for different concentrations of gelatin are shown in Figure 4. The relaxation times, relaxivities, and permittivity measurements for gelatin are summarized in Table 4.

The T_1_ and T_2_ relaxation rates (R_1_ and R_2_, respectively) as a function of polyethylene glycol (PEG) concentration are shown in Figures 5a and 5b The T_1_ and T_2_ relaxivities (r_1_ and r_2_, respectively) as a function of field strength are shown in Figures 5c and 5d.

The dielectric strengths and electrical conductivities for different concentrations of PEG are shown in Figure 6. The relaxation times, relaxivities, and permittivity measurements for PEG are summarized in Table 5.

The T_1_ and T_2_ relaxation rates (R_1_ and R_2_, respectively) as a function of polyvinyl alcohol (PVA) concentration are shown in Figures 7a and 7b The T_1_ and T_2_ relaxivities (r_1_ and r_2_, respectively) as a function of field strength are shown in Figures 7c and 7d.

The dielectric strengths and electrical conductivities for different concentrations of PVA are shown in Figure 8. The relaxation times, relaxivities, and permittivity measurements for PVA are summarized in Table 6.

The T_1_ and T_2_ relaxation rates (R_1_ and R_2_, respectively) as a function of polyvinylpyrrolidone (PVP) concentration are shown in Figures 9a and 9b The T_1_ and T_2_ relaxivities (r_1_ and r_2_, respectively) as a function of field strength are shown in Figures 9c and 9d.

The dielectric strengths and electrical conductivities for different concentrations of PVP are shown in Figure 10. The relaxation times, relaxivities, and permittivity measurements for PVP are summarized in Table 7.

The T_1_ and T_2_ relaxation rates (R_1_ and R_2_, respectively) as a function of sodium alginate concentration are shown in Figures 11a and 11b The T_1_ and T_2_ relaxivities (r_1_ and r_2_, respectively) as a function of field strength are shown in Figures 11c and 11d.

The dielectric strengths and electrical conductivities for different concentrations of sodium alginate are shown in Figure 12. The relaxation times, relaxivities, and permittivity measurements for sodium alginate are summarized in Table 8.

The T_1_ and T_2_ relaxation rates (R_1_ and R_2_, respectively) as a function of sodium polyacrylate concentration are shown in Figures 13a and 13b The T_1_ and T_2_ relaxivities (r_1_ and r_2_, respectively) as a function of field strength are shown in Figures 13c and 13d.

The dielectric strengths and electrical conductivities for different concentrations of sodium polyacrylate are shown in Figure 14. The relaxation times, relaxivities, and permittivity measurements for sodium polyacrylate are summarized in Table 9.

The T_1_ and T_2_ relaxation rates (R_1_ and R_2_, respectively) as a function of xanthan gum concentration are shown in Figures 15a and 15b The T_1_ and T_2_ relaxivities (r_1_ and r_2_, respectively) as a function of field strength are shown in Figures 15c and 15d.

The dielectric strengths and electrical conductivities for different concentrations of xanthan gum are shown in Figure 16. The relaxation times, relaxivities, and permittivity measurements for xanthan gum are summarized in Table 10.

### Paramagnetic Salts:

The T_1_ and T_2_ relaxation rates (R_1_ and R_2_, respectively) as a function of copper sulfate concentration are shown in Figures 17a and 17b The T_1_ and T_2_ relaxivities (r_1_ and r_2_, respectively) as a function of field strength are shown in Figures 17c and 17d.

The dielectric strengths and electrical conductivities for different concentrations of copper sulfate are shown in Figure 18. The relaxation times, relaxivities, and permittivity measurements for copper sulfate are summarized in Table 11.

The T_1_ and T_2_ relaxation rates (R_1_ and R_2_, respectively) as a function of manganese nitrate concentration are shown in Figures 19a and 9b The T_1_ and T_2_ relaxivities (r_1_ and r_2_, respectively) as a function of field strength are shown in Figures 19c and 19d.

The dielectric strengths and electrical conductivities for different concentrations of manganese nitrate are shown in Figure 20. The relaxation times, relaxivities, and permittivity measurements for manganese nitrate are summarized in Table 12.

The T_1_ and T_2_ relaxation rates (R_1_ and R_2_, respectively) as a function of nickel chloride concentration are shown in Figures 21a and 21b The T_1_ and T_2_ relaxivities (r_1_ and r_2_, respectively) as a function of field strength are shown in Figures 21c and 21d.

The dielectric strengths and electrical conductivities for different concentrations of nickel chloride are shown in Figure 22. The relaxation times, relaxivities, and permittivity measurements for nickel chloride are summarized in Table 13.

### Oils:

The T_1_ and T_2_ relaxation rates (R_1_ and R_2_, respectively) as a function of field strength are shown in Figures 23a and 23b for canola, castor, and grapeseed oils. Deionized water and 0.9% (normal) saline are shown for comparison. The relaxation times are documented in Table 14. The permittivity results are shown in Table 15.

The viscosities measured at 22^0^C were: canola oil (70.54 +/− 0.09 mm^2^/s), castor oil (826.62 +/− 7.55 mm^2^/s),and grapeseed oil (61.42 +/− 0.25 mm^2^/s).^27^ Proton densities relative to water were 0.96 (castor oil), 1.02 (canola oil), and 0.93 (grapeseed oil).

## 4. CONCLUSIONS

In principle, T_1_ of tissue should increase with field strength while T_2_ should not vary with field strength.^28,29^ However, the T_1_ of NiCl_2_ did not rise with field strength. We observed T_2_ decreasing with increasing field strength in PEG, PVA, sodium polyacrylate, and Mn(NO_3_)_2_. T_2_ increased with field strength in CuSO_4_.

T_1_ relaxivities (r_1_) may drop with rising field strength.^30^ However, r_1_ remained constant in PEG and PVP, and rose for NiCl_2_. T_2_ relaxivities (r_2_) typically remain constant or rise with rising field strength. Yet r_2_ dropped for CuSO_4_. The 0.35 T relaxivities for Cu^+2^ and Ni^+2^ were similar to relaxivities at 0.5 T.^31^

Phantoms for measuring RF heating require electrical conductivities similar to in vivo (0–1.5 S/m). Saline's dielectric decreases and conductivity increases with salinity.^32^ Addition of paramagnetic electrolytes also increases electrical conductivities. Mn(NO_3_)_2_ had significantly higher relaxation rates compared to CuSO_4_ and NiCl_2._ Our 3 T relaxivity results were similar to those from a 3 T study.^15^ Our 0.35 T relaxation times and relaxivities for Ni^+2^ and Cu^+2^ were similar to 0.5 T values from an earlier study.^31^ Caution must be used with the paramagnetic electrolytes since they can be corrosive (Cu^+2^, Mn^+2,^ and Ni^+2^), neurotoxic (Mn^+2^), and carcinogenic (Cu^+2^ and Ni^+2^). Our 1.5 T CuSO_4_ relaxation times were consistent with an earlier study.^33^

QA for high field strengths (B_0_>1.5 T) requires low dielectric phantoms to avoid dielectric artifacts. Oils typically have low dielectrics and electrical conductivities.^34^ $\varepsilon_{s}$ was slowly varying for hydrogel concentration except for PVP which can drop $\varepsilon_{s}$ to <40.^35,36^ PVP also has a sharp dominant peak that can minimize chemical shift artifacts.

Multiple compartment phantom materials were modeled to adjust T_1_ and T_2_ to be similar to specific tissues.^7 37–40^ Conductive oils that combine polymers (e.g., polypropylene glycol) and metal (e.g., zinc oxide) may present an opportunity.

Preparation of homogeneous hydrogels was a challenge in this study. Originally, the Nambu freezing and thawing method was used to prepare the PVA gel at concentrations ranging from 10% to 30% (w/w).^5^ However, the resulting gel was too firm for the purpose of this experiment, so the simple aqueous solution described was prepared at lower concentrations instead. Nevertheless, the heterogeneity of the hydrogels often resulted in high variance in the relaxation rates. Combinations of materials have been published.^17,41^ Longevities of <3 months are typically assumed.^25^

Synthetic polymers are more desirable in phantoms than organic polymers since the synthetic polymers are more resistant to organic growth.^42^ The paramagnetic electrolytes tend to prevent microorganisms from growing in the phantoms. They also increase the salinity and conductivity of the resulting liquid. However, electrolytes can impact gelling so higher concentrations of the gelling agent may be necessary.^43^

PVP is used in diffusion phantom manufacturing.^8^ Our T_2_ values for PVP were significantly higher than other studies.^44^ Our sodium alginate results were similar to relaxation times measured at 2.35 T.^45^ Our measured relaxivities for PEG were small for the tested range.

In summary, sodium polyacrylate, PVP, and PEG hydrogels were challenging for generating homogeneous phantoms. Sodium alginate, Miller's LB agar, and xanthan gum had good properties although they are vulnerable to biological growth. Generally, hydrogels have a limited life while paramagnetic electrolytes and oils may have a long life.

All three oils (canola, castor, and grapeseed) were good candidates for low dielectric (ε_s_<10) phantoms (e.g., high B_0_) with low conductivities. Mn(NO_3_)_2_ had the highest relaxivities of the paramagnetic electrolytes tested.

Our study is unique since it covers variations of phantom materials versus concentration and field strength. The main limitation is we characterized single (oil) or duplex (water plus gel or electrolyte) materials.

## Supplementary Material

Supplement 1

## Figures and Tables

**FIGURE 1:**
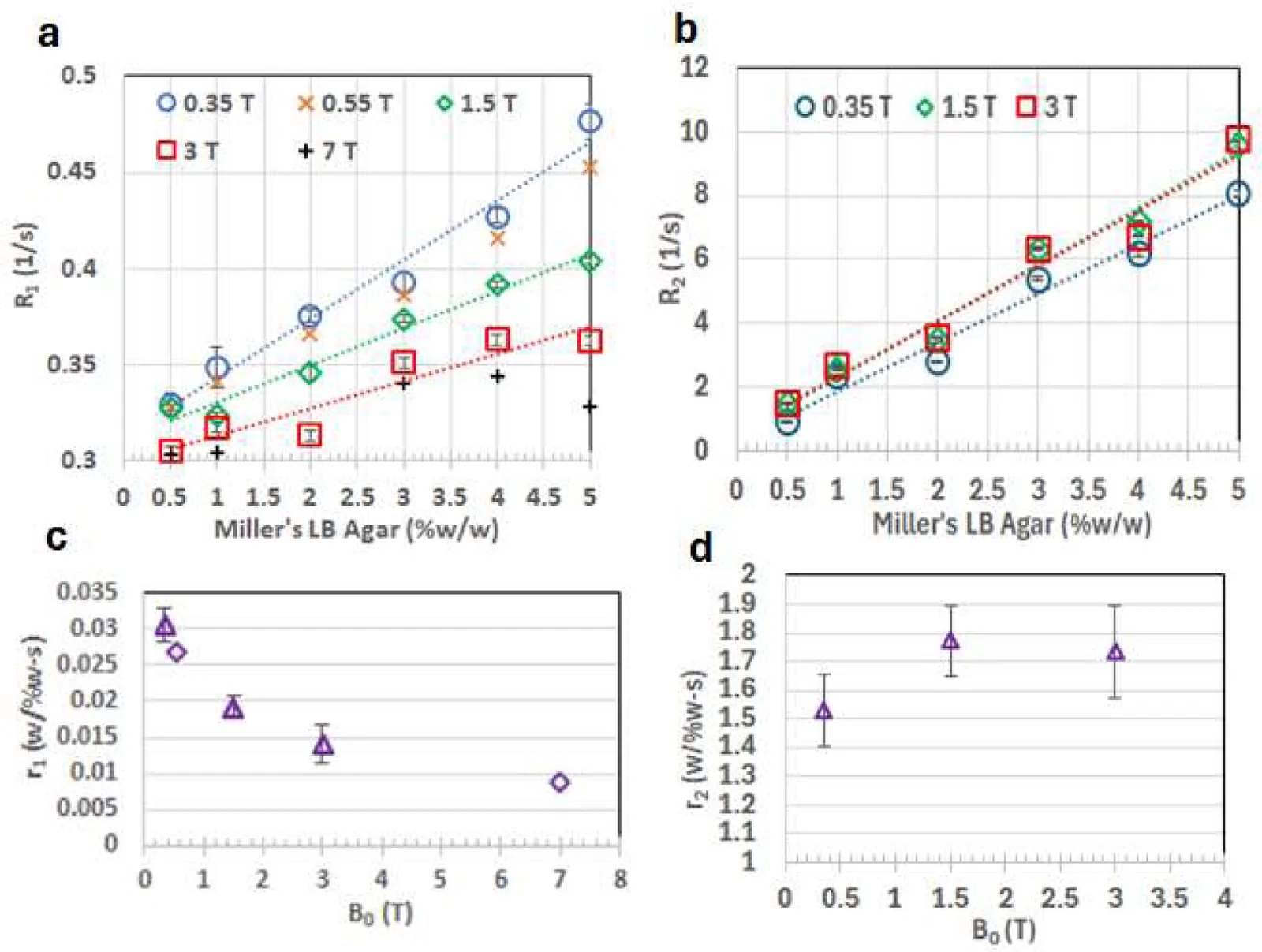
Relaxation rates (R_1_, R_2_) for different concentrations of Miller's LB agar in water: a) R_1_ and b) R_2_, and relaxivities (r_1_, r_2_) as a function of field strength for c) r_1_ and d) r_2_. Miller's LB agar is a mixture including agar (32% w/w). R_1_ (×, **+**) and r_1_ (◊) interpolations/extrapolations are included for 0.55 T and 7 T. Note the R_1_ extrapolated for 7 T and concentration 5% w/w is lower than R_1_ for 4% w/w suggesting that the extrapolation is false.

**FIGURE 2:**
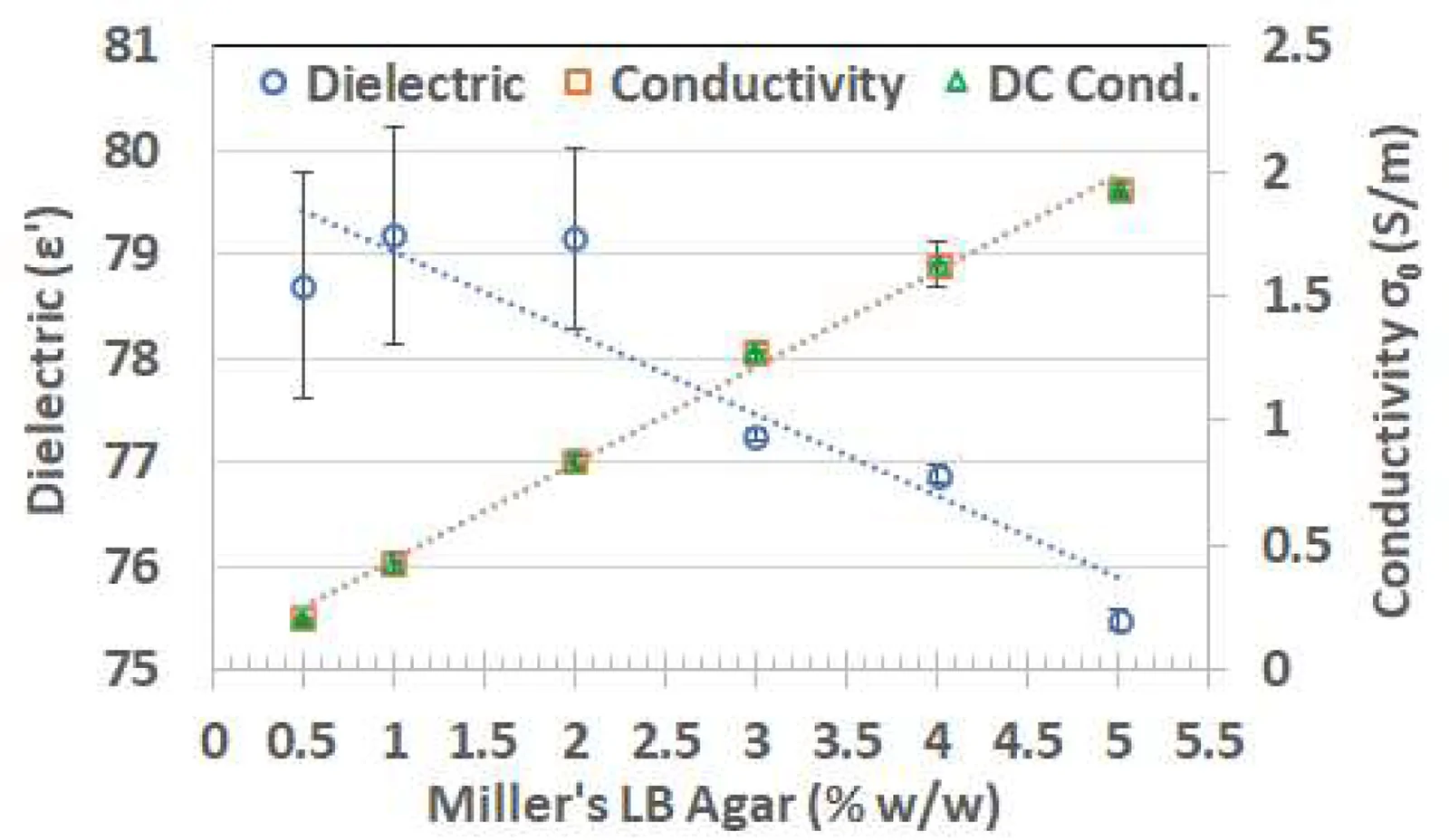
Static dielectric constant ε_s_ (○, left axis), static electrical conductivity σ_s_ (□) and DC conductivity σ_DC_ from the conductivity meter (Δ) are shown for different concentrations of Miller's LB agar in water. σ_s_ and σ_DC_ were highly correlated (r=0.997). ε_s_ and σ_s_ were highly correlated (r=−0.912).

**FIGURE 3:**
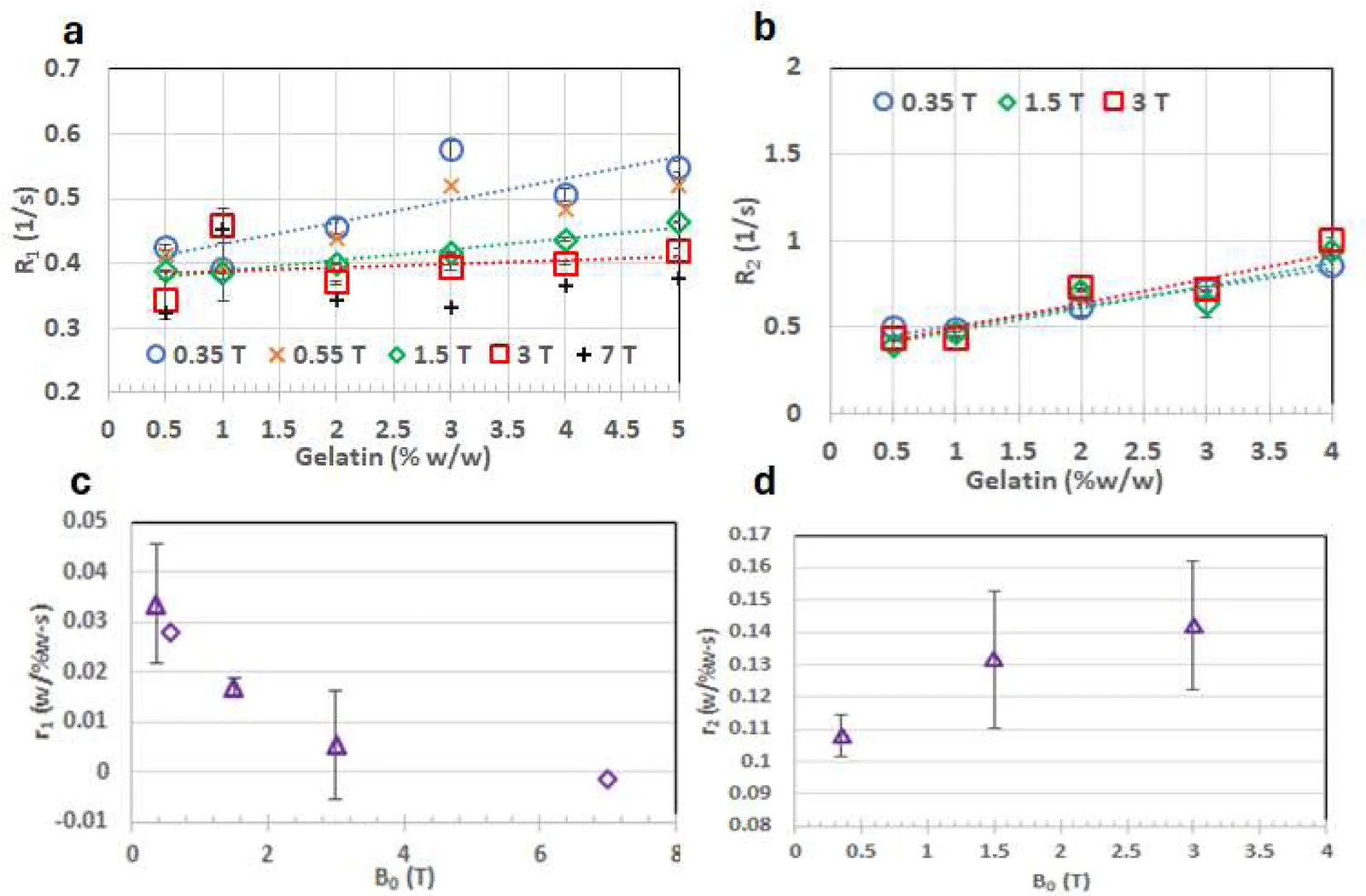
Relaxation rates for different concentrations of gelatin: a) R_1_ and b) R_2,_ and gelatin relaxivities vs. field strength for c) r_1_ and d) r_2_. R_1_ (×, **+**) and r_1_ (◊) interpolations/extrapolations are included for 0.55 T and 7 T.

**FIGURE 4:**
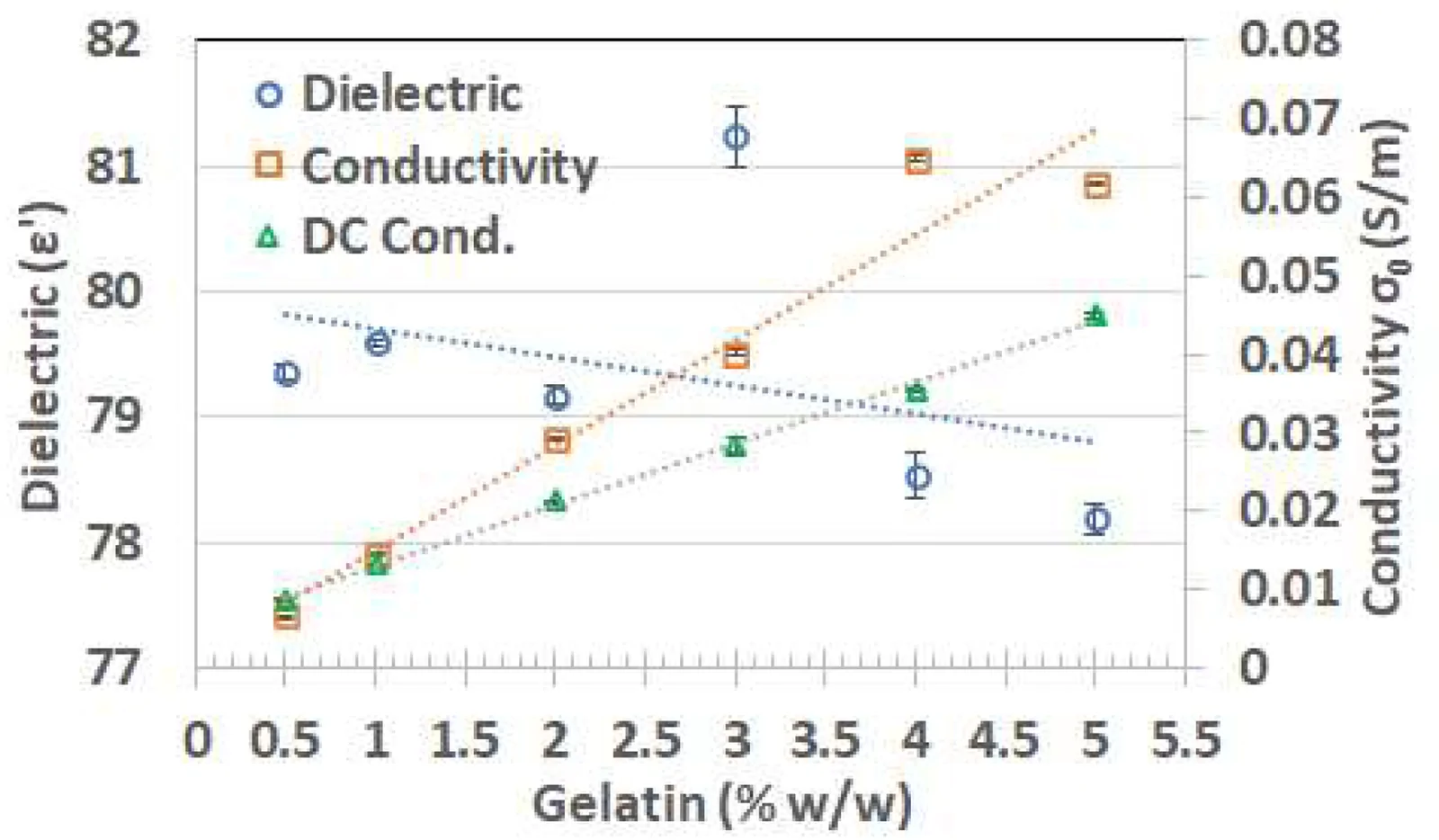
Static dielectric constant ε_s_ (○, left axis), static electrical conductivity σ_s_ (□) and DC conductivity σ_DC_ from the conductivity meter (Δ) are shown for different concentrations of gelatin in water. σ_s_ and σ_DC_ were highly correlated (r=0.966).

**FIGURE 5:**
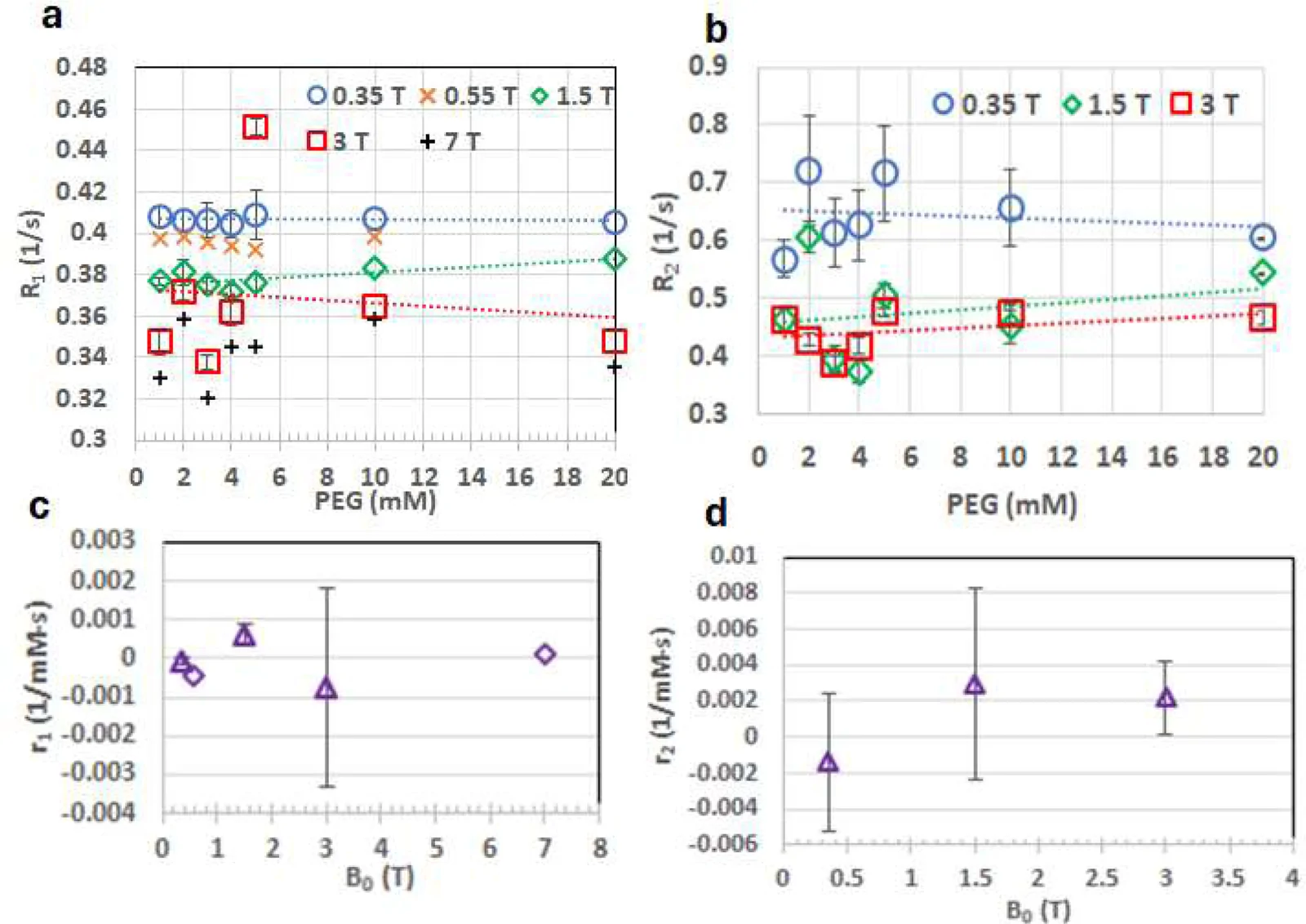
Relaxation rates for different concentrations of polyethylene glycol (PEG): a) R_1_ and b) R_2,_ and PEG relaxivities vs. field strength for c) r_1_ and d) r_2_. R_1_ (×, **+**) and r_1_ (◊) interpolations/extrapolations are included for 0.55 T and 7 T.

**FIGURE 6:**
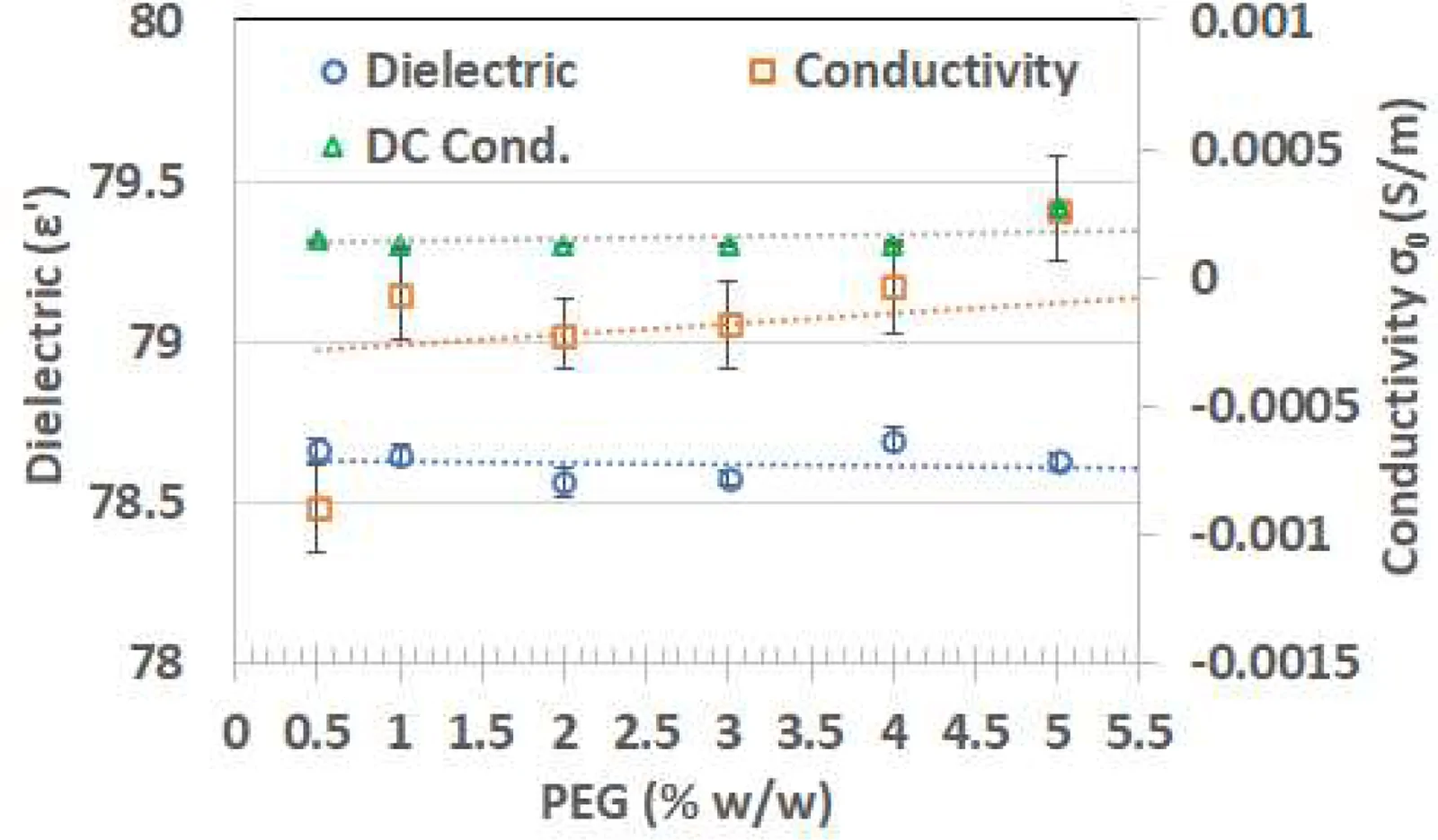
Static dielectric constant ε_s_ (○, left axis), static electrical conductivity σ_s_ (□) and DC conductivity σ_DC_ from the conductivity meter (Δ) are shown for different concentrations of PEG in water. σ_s_ and σ_DC_ were correlated (r=0.682).

**FIGURE 7:**
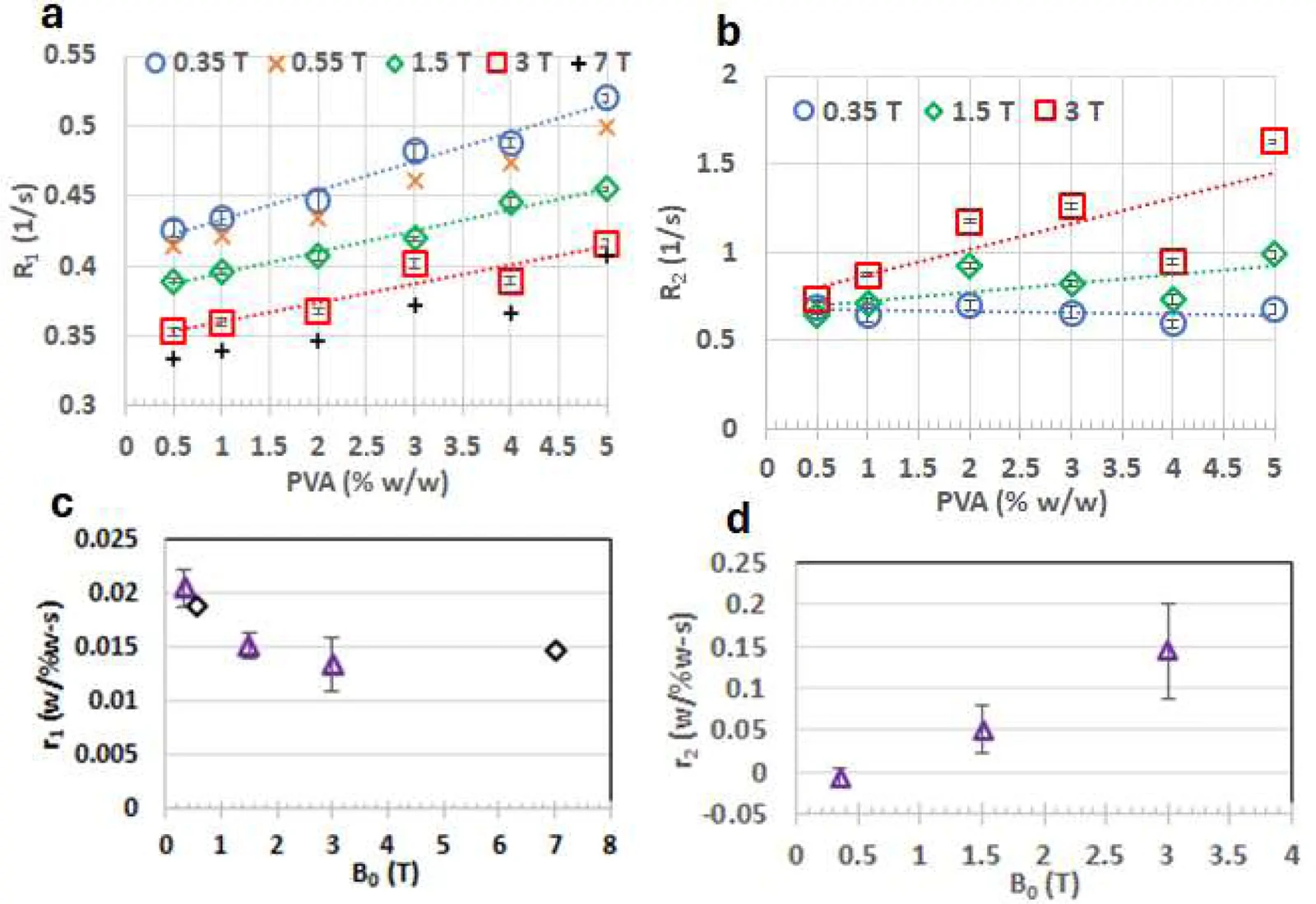
Relaxation rates for different concentrations of polyvinyl alcohol (PVA): a) R_1_ and b) R_2,_ and PVA relaxivities vs. field strength for c) r_1_ and d) r_2_. R_1_ (×, **+**) and r_1_ (◊) interpolations/extrapolations are included for 0.55 T and 7 T.

**FIGURE 8:**
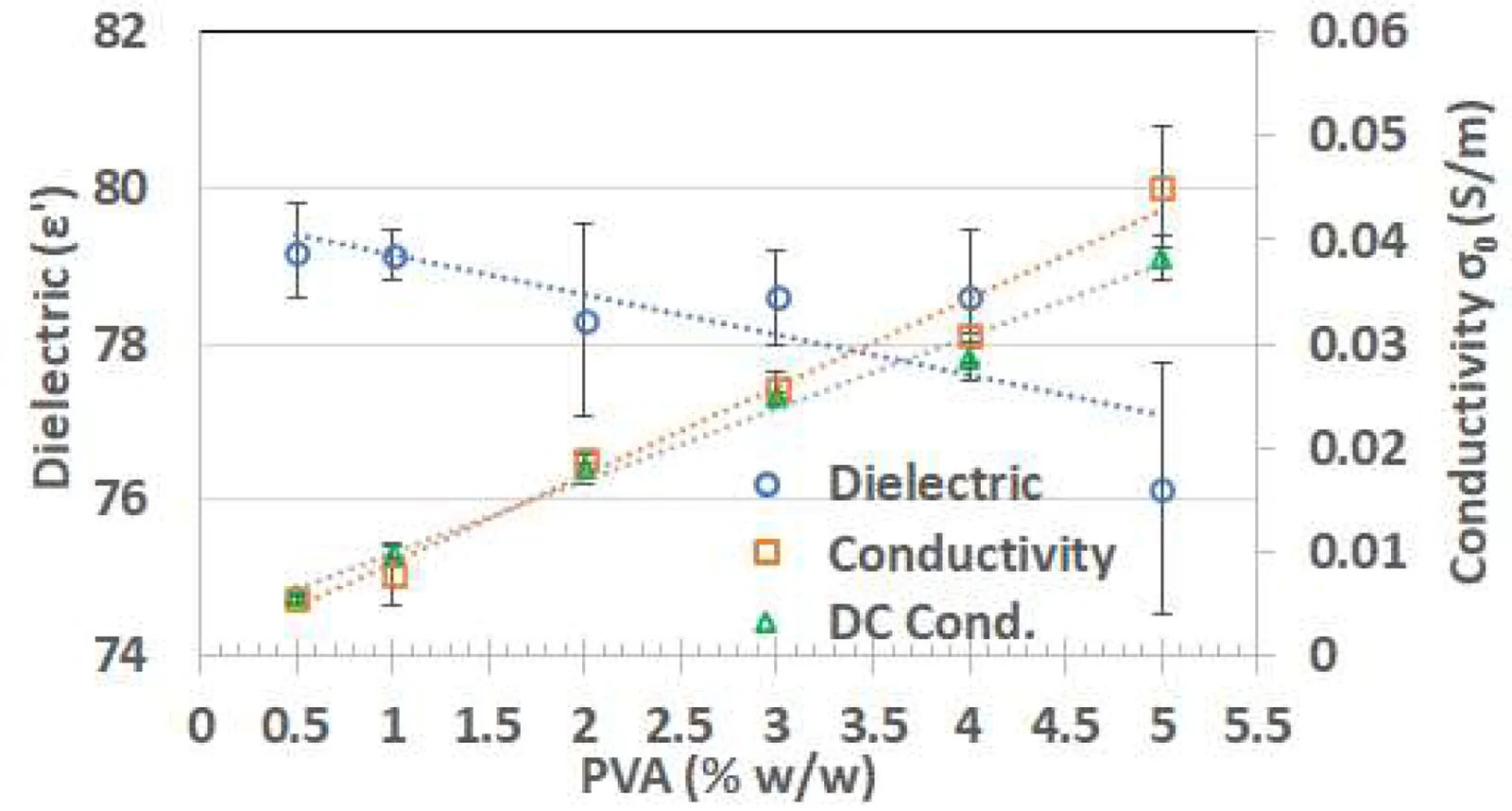
Static dielectric constant ε_s_ (○, left axis), static electrical conductivity σ_s_ (□) and DC conductivity σ_DC_ from the conductivity meter (Δ) are shown for different concentrations of PVA in water. σ_s_ and σ_DC_ were highly correlated (r=0.996). ε_s_ and σ_s_ were highly correlated (r=−0.870).

**FIGURE 9:**
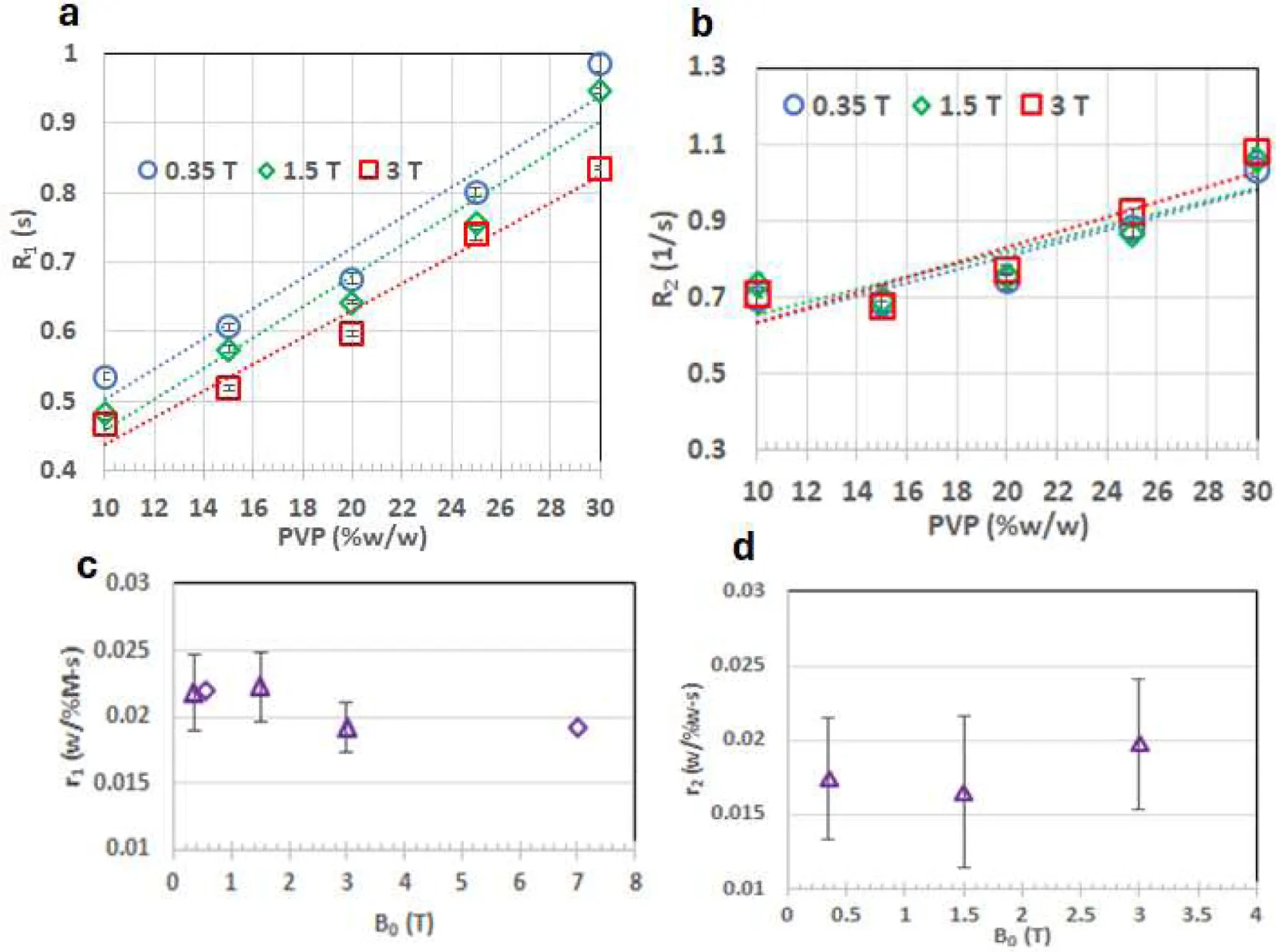
Relaxation rates for different concentrations of polyvinylpyrrolidone (PVP): a) R_1_ and b) R_2,_ and PVP relaxivities vs. field strength for c) r_1_ and d) r_2_. R_1_ (×, **+**) and r_1_ (◊) interpolations/extrapolations are included for 0.55 T and 7 T.

**FIGURE 10:**
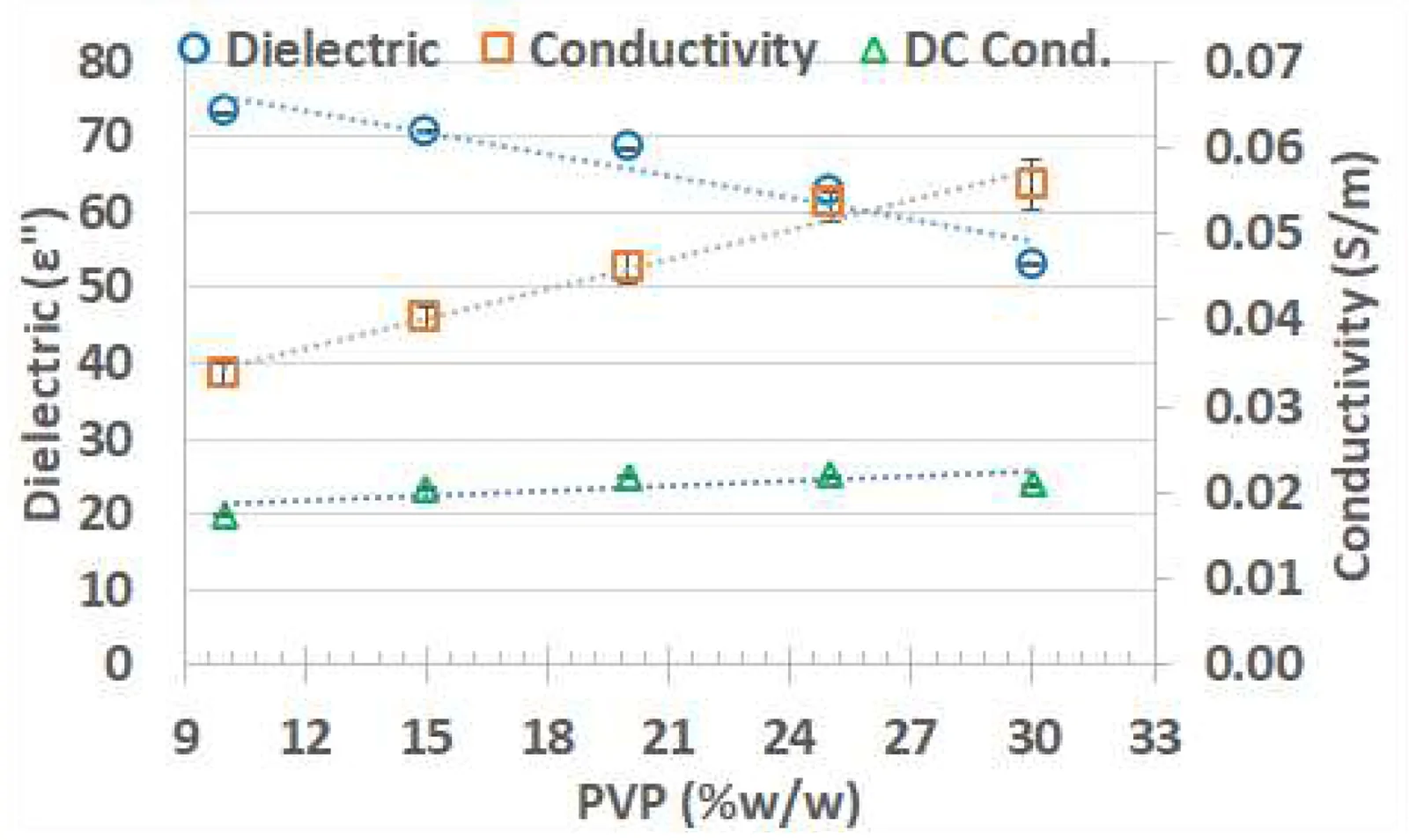
Static dielectric constant ε_s_ (○, left axis), static electrical conductivity σ_s_ (□) and DC conductivity σ_DC_ from the conductivity meter (Δ) are shown for different concentrations of PVP in water. σ_s_ and σ_DC_ were highly correlated (r=−0.811) but significantly different (p<0.01). ε_s_ and σ_s_ were highly correlated (r=−0.811).

**FIGURE 11:**
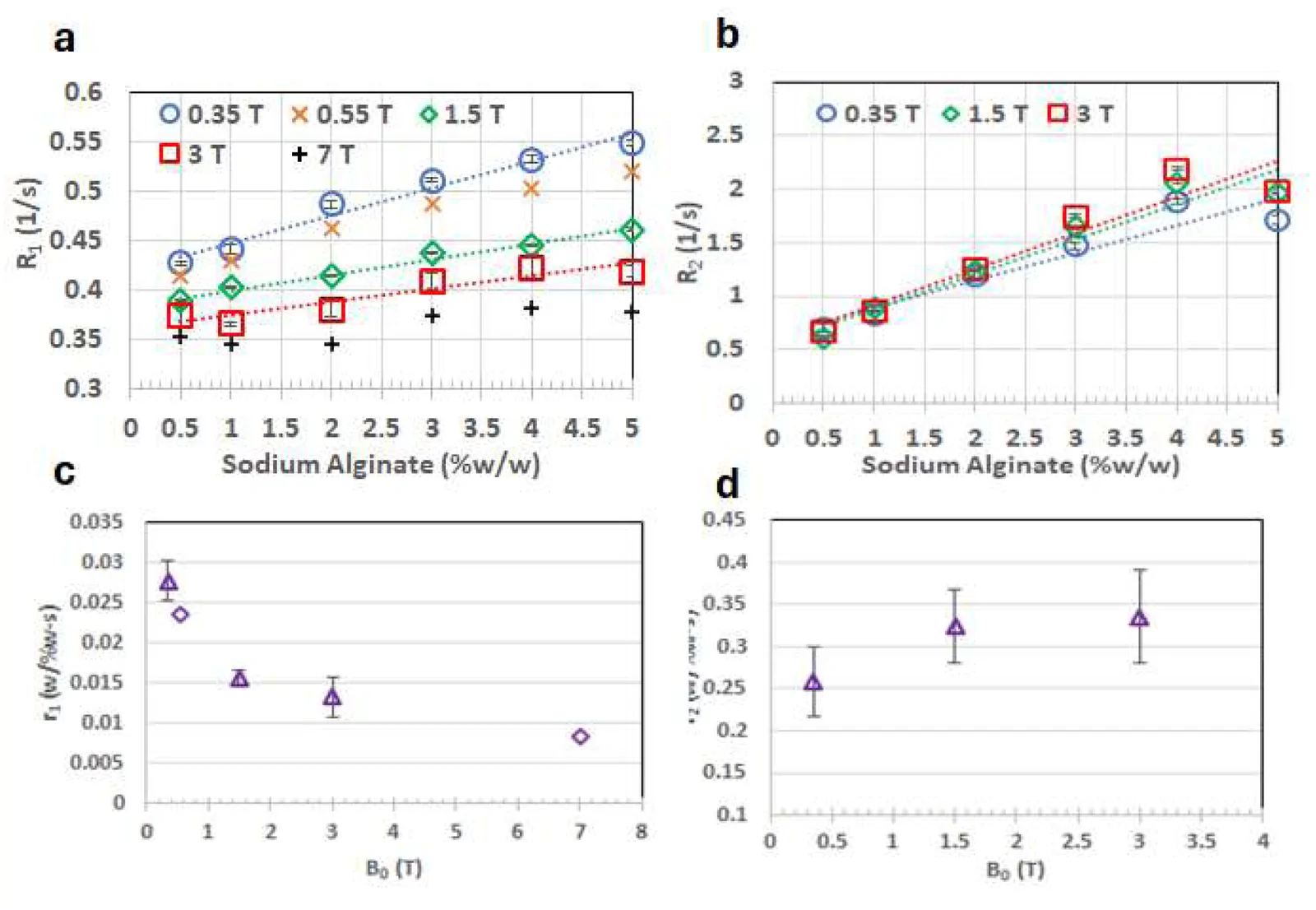
Relaxation rates for different concentrations of sodium alginate: a) R_1_ and b) R_2,_ and sodium alginate relaxivities vs. field strength for c) r_1_ and d) r_2_. R_1_ (×, **+**) and r_1_ (◊) interpolations/extrapolations are included for 0.55 T and 7 T.

**FIGURE 12:**
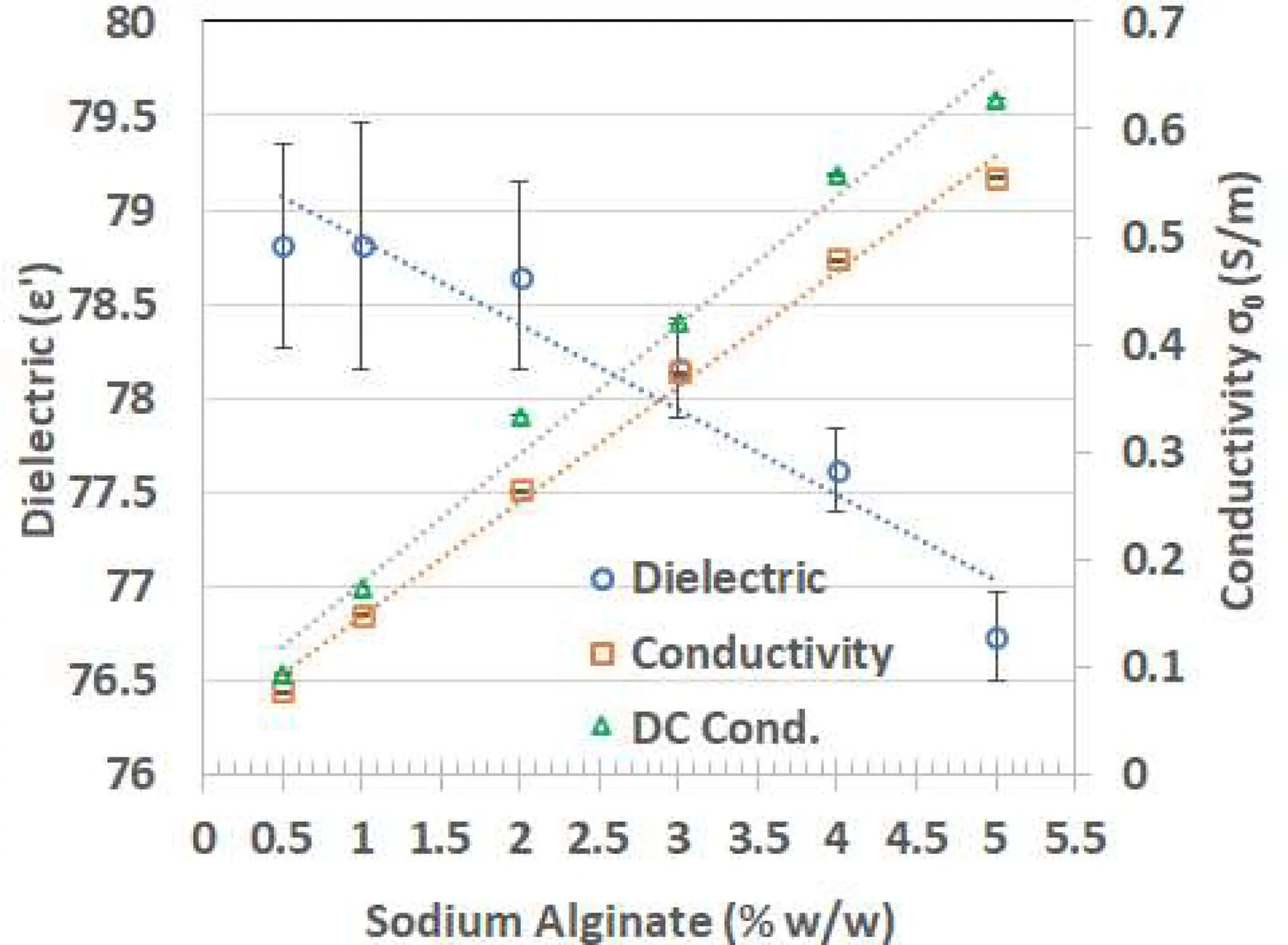
Static dielectric constant ε_s_ (○, left axis), static electrical conductivity σ_s_ (□) and DC conductivity σ_DC_ from the conductivity meter (Δ) are shown for different concentrations of sodium alginate in water. σ_s_ and σ_DC_ were highly correlated (r=0.998) and significantly different (p<0.01). ε_s_ and σ_s_ were highly correlated (r=−0.931).

**FIGURE 13:**
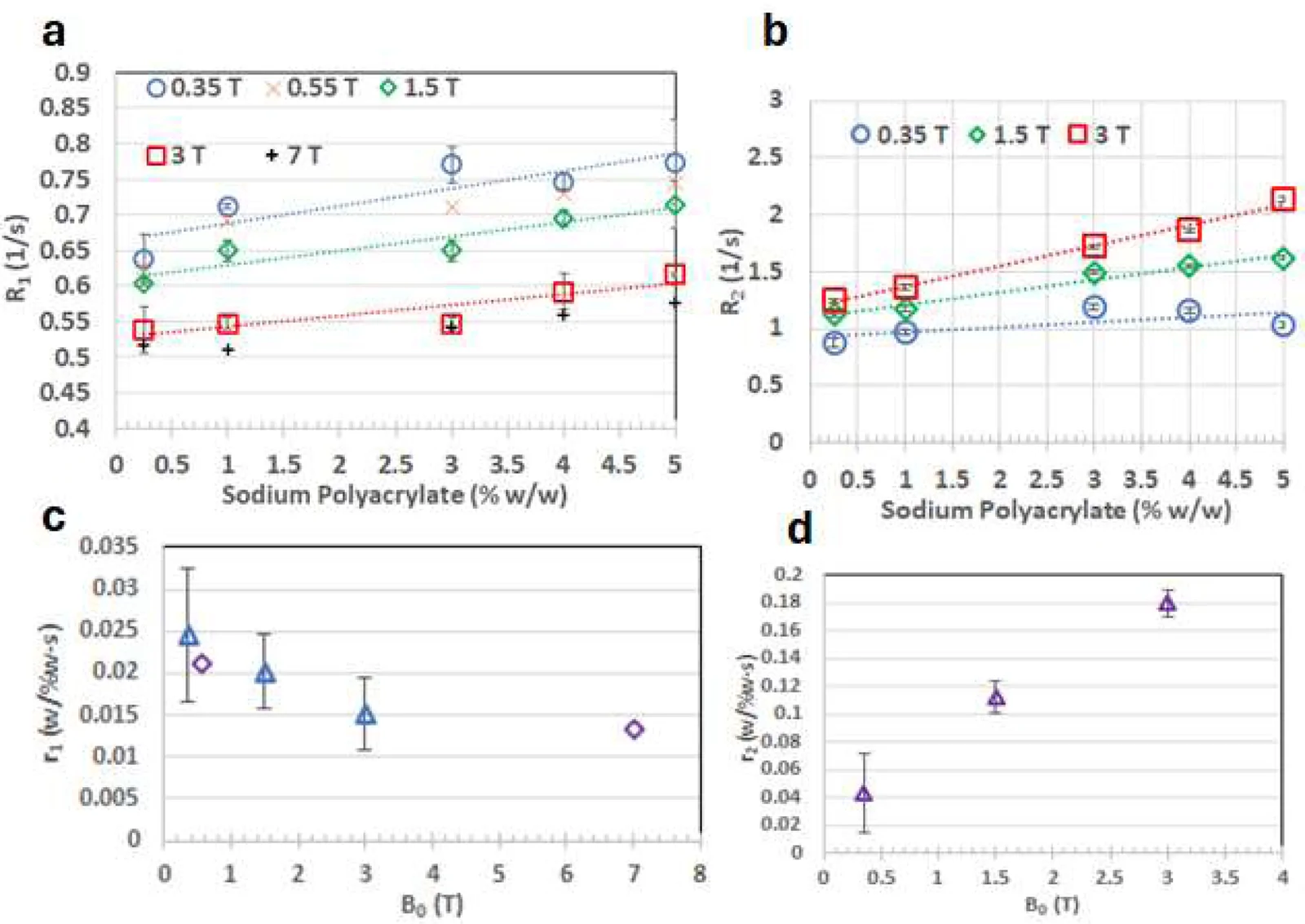
Relaxation rates for different concentrations of sodium polyacrylate: a) R_1_ and b) R_2_, and sodium polyacrylate relaxivities vs. field strength for c) r_1_ and d) r_2_. R_1_ (×, **+**) and r_1_ (◊) interpolations/extrapolations are included for 0.55 T and 7 T.

**FIGURE 14:**
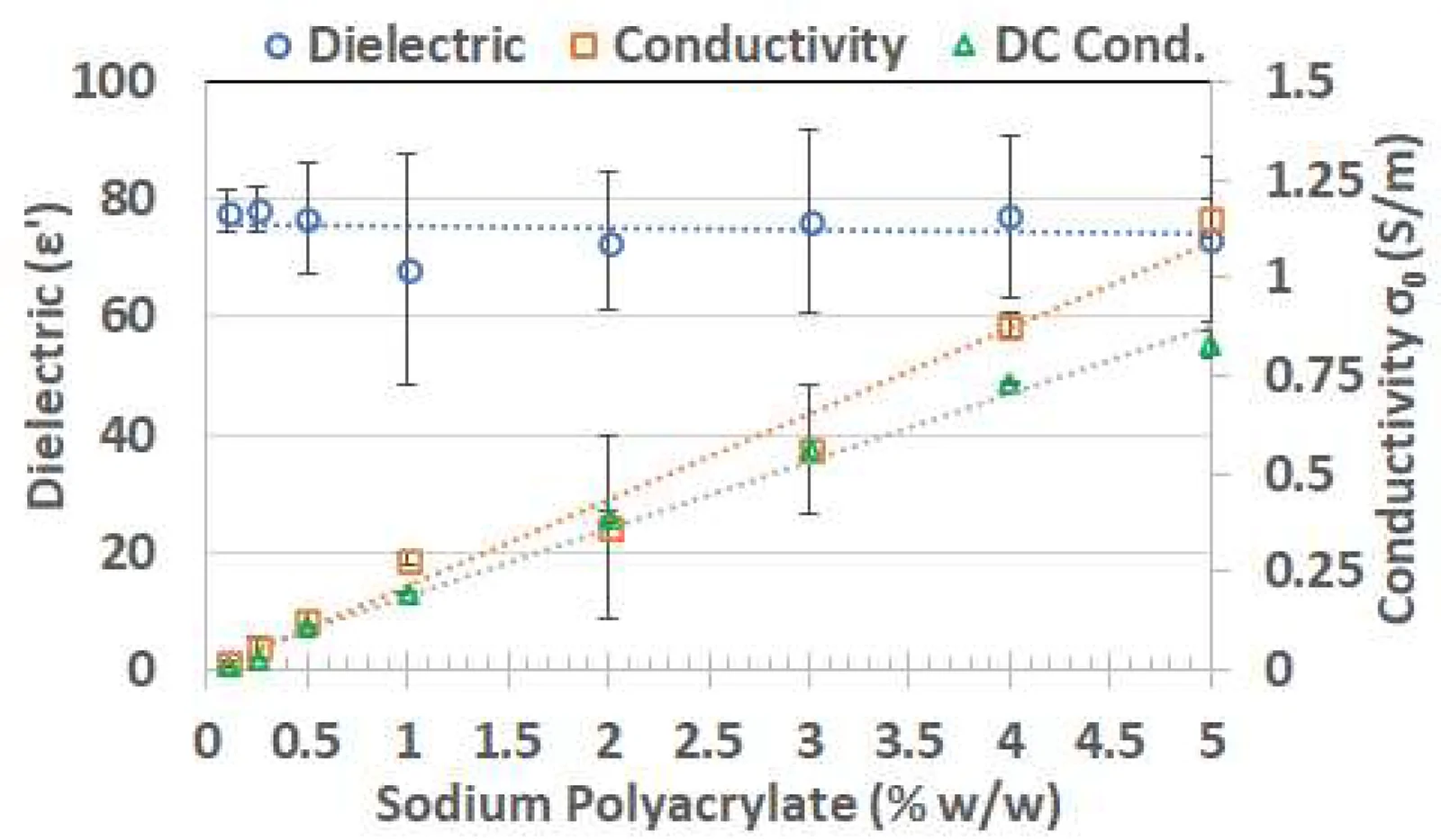
Static dielectric constant ε_s_ (○, left axis), static electrical conductivity σ_s_ (□) and DC conductivity σ_DC_ from the conductivity meter (Δ) are shown for different concentrations of sodium polyacrylate in water. σ_s_ and σ_DC_ were highly correlated (r=0.921). The static dielectric did not vary over the tested concentrations.

**FIGURE 15:**
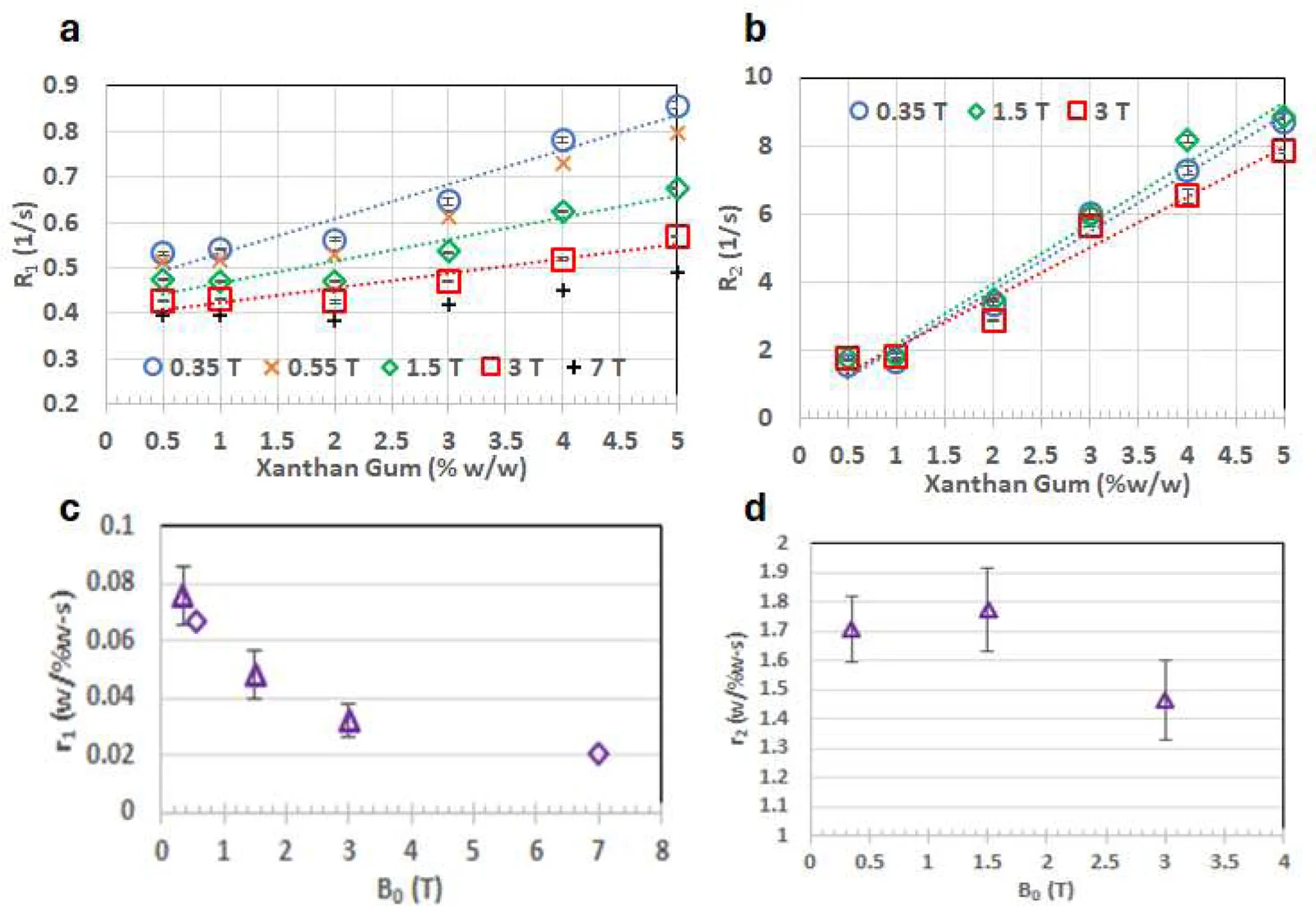
Relaxation rates for different concentrations of xanthan gum: a) R_1_ and b) R_2_, and xanthan gum relaxivities vs. field strength for c) r_1_ and d) r_2_. R_1_ (×, **+**) and r_1_ (◊) interpolations/extrapolations are included for 0.55 T and 7 T.

**FIGURE 16:**
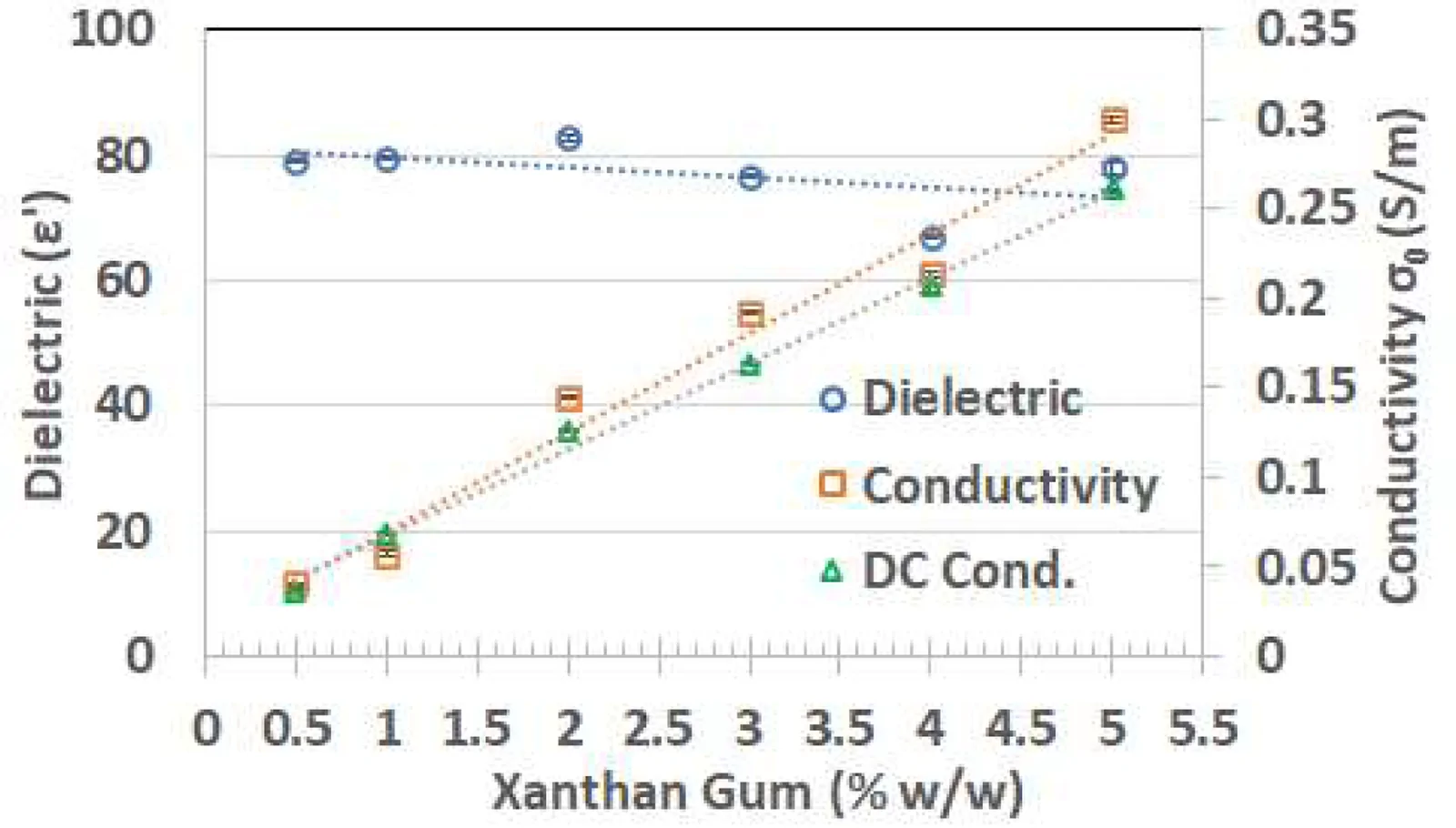
Static dielectric constant ε_s_ (○, left axis), static electrical conductivity σ_s_ (□) and DC conductivity σ_DC_ from the conductivity meter (Δ) are shown for different concentrations of xanthan gum in water. σ_s_ and σ_DC_ were highly correlated (r=0.992).

**FIGURE 17:**
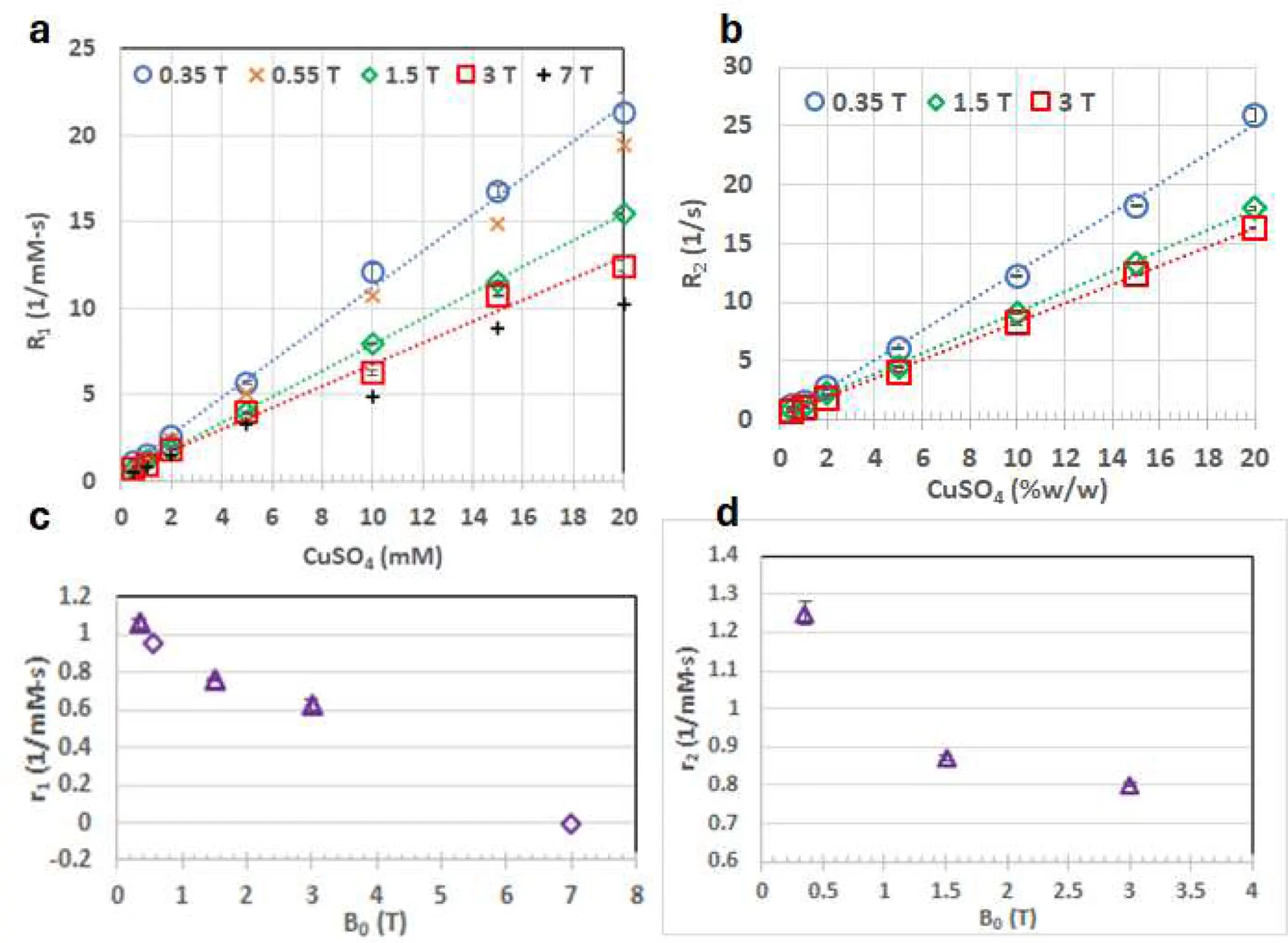
Relaxation rates for different concentrations of CuSO_4_: a) R_1_ and b) R_2,_ and CuSO_4_ relaxivities vs. field strength for c) r_1_ and d) r_2_. R_1_ (×, **+**) and r_1_ (◊) interpolations/extrapolations are included for 0.55 T and 7 T.

**FIGURE 18:**
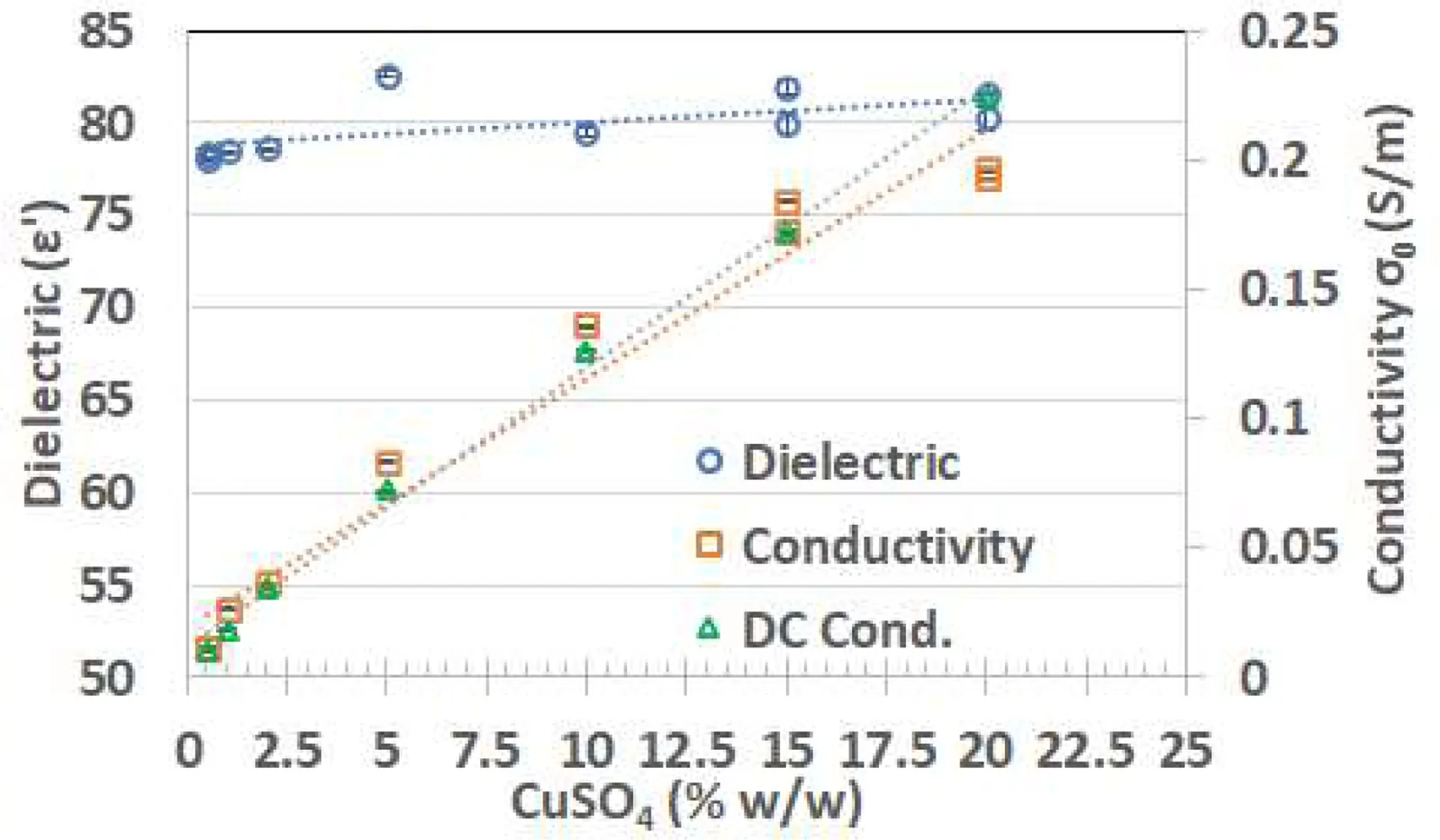
Static dielectric constant ε_s_ (○, left axis), static electrical conductivity σ_s_ (□) and DC conductivity σ_DC_ from the conductivity meter (Δ) are shown for different concentrations of CuSO_4_ in water. σ_s_ and σ_DC_ were highly correlated (r=0.985). ε_s_ and σ_s_ were correlated (r=−0.653).

**FIGURE 20:**
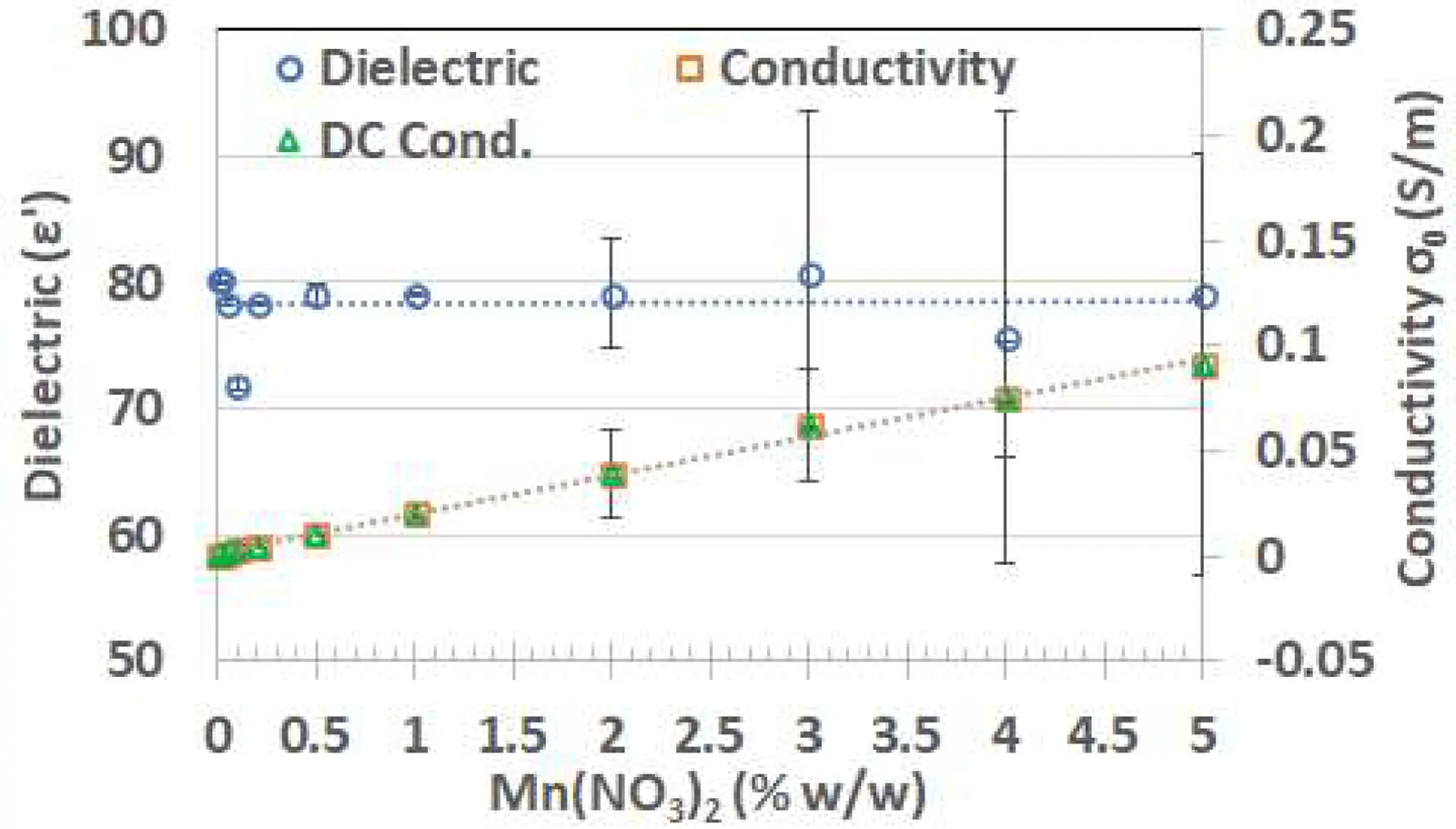
Static dielectric constant ε_s_ (○, left axis), static electrical conductivity σ_s_ (□) and DC conductivity σ_DC_ from the conductivity meter (Δ) are shown for different concentrations of Mn(NO_3_)_2_ in water. σ_s_ and σ_DC_ were highly correlated (r=0.985).

**FIGURE 21:**
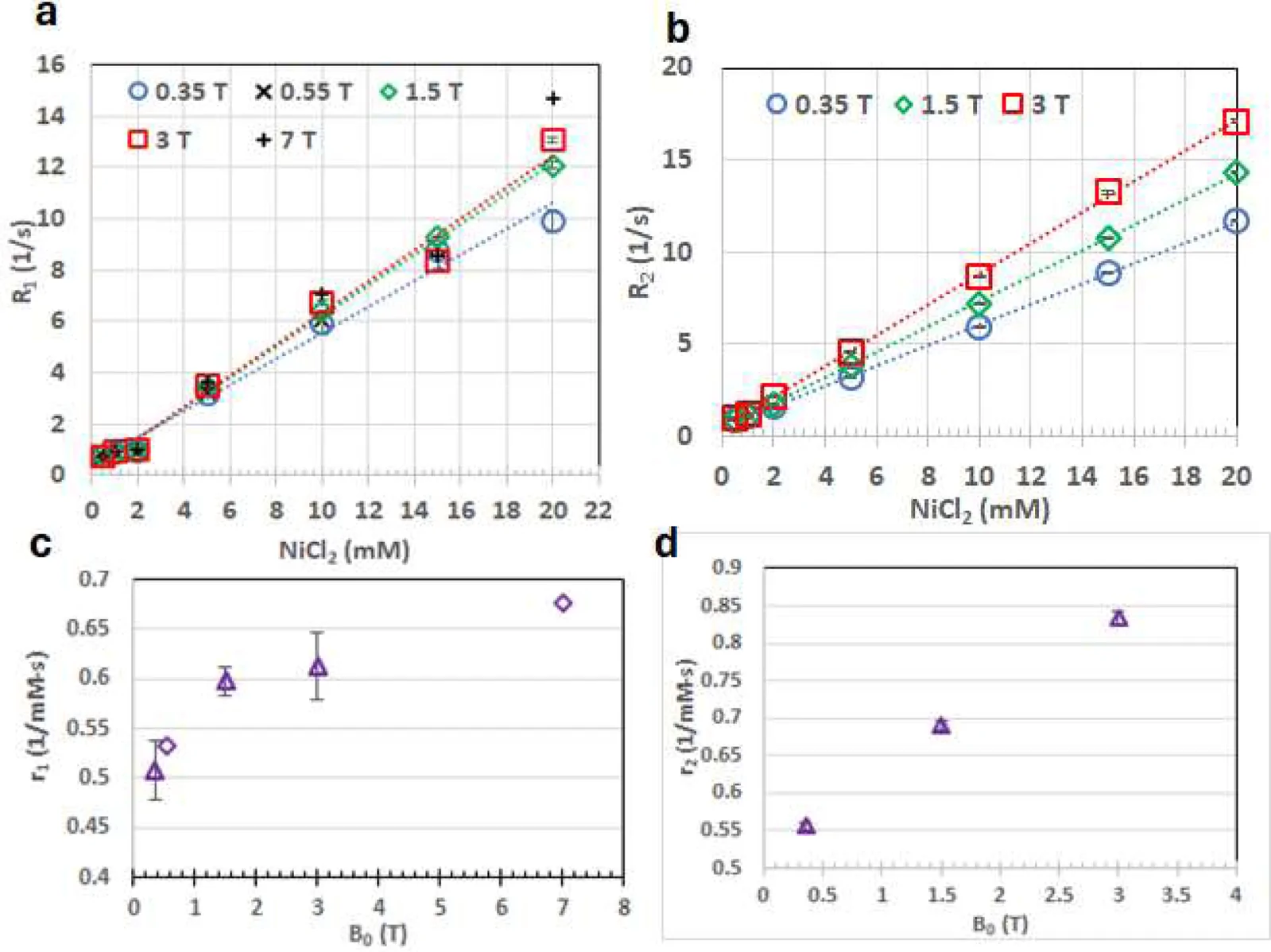
Relaxation rates for different concentrations of NiCl_2_: a) R_1_ and b) R_2,_ and NiCl_2_ relaxivities vs. field strength for c) r_1_ and d) r_2_. R_1_ (×, **+**) and r_1_ (◊) interpolations/extrapolations are included for 0.55 T and 7 T.

**FIGURE 22:**
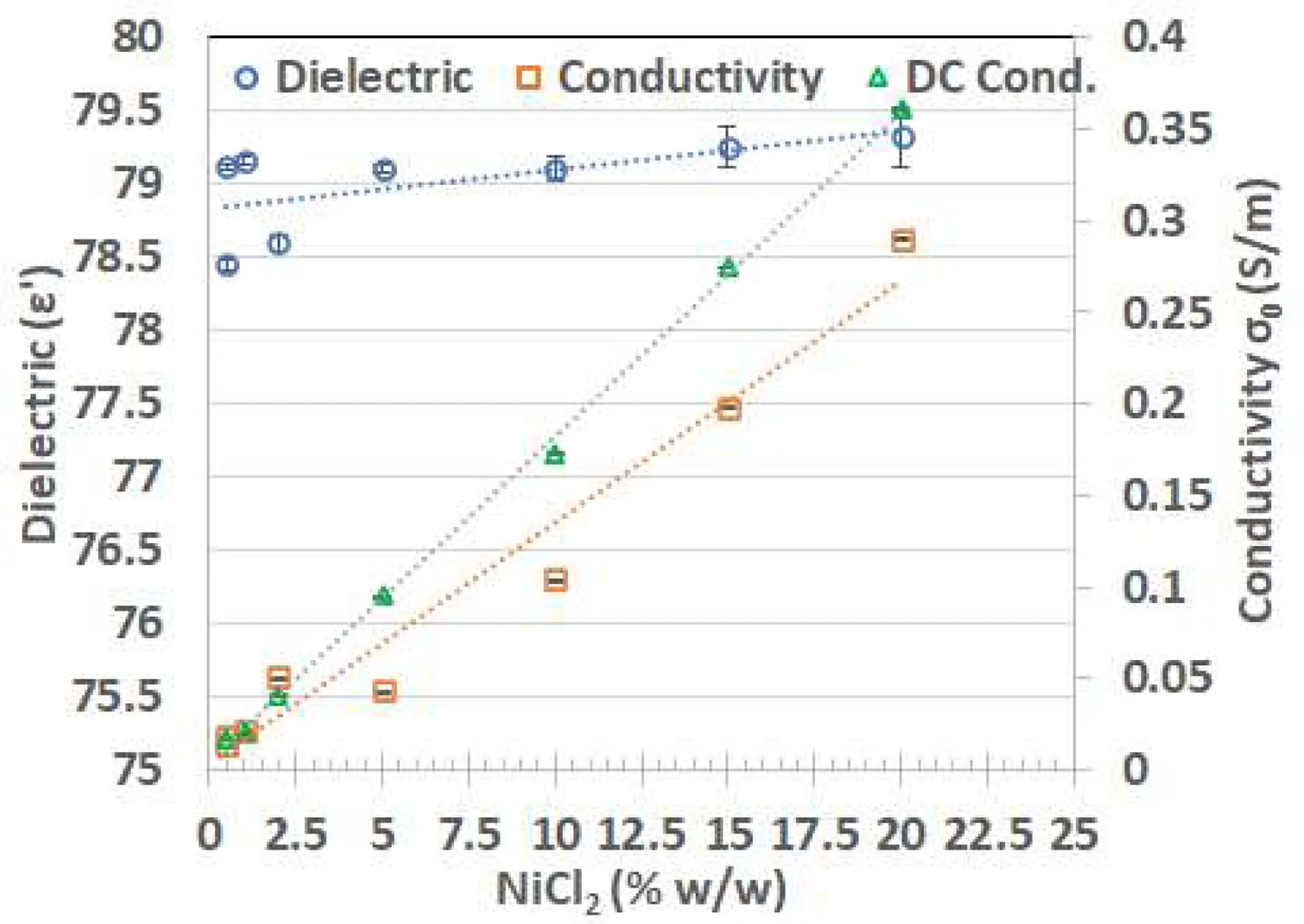
Static dielectric constant ε_s_ (○, left axis), static electrical conductivity σ_s_ (□) and DC conductivity σ_DC_ from the conductivity meter (Δ) are shown for different concentrations of NiCl_2_ in water. σ_s_ and σ_DC_ were highly correlated (r=0.981) but significantly different. ε_s_ and σ_s_ were correlated (r=−0.653).

**FIGURE 23:**
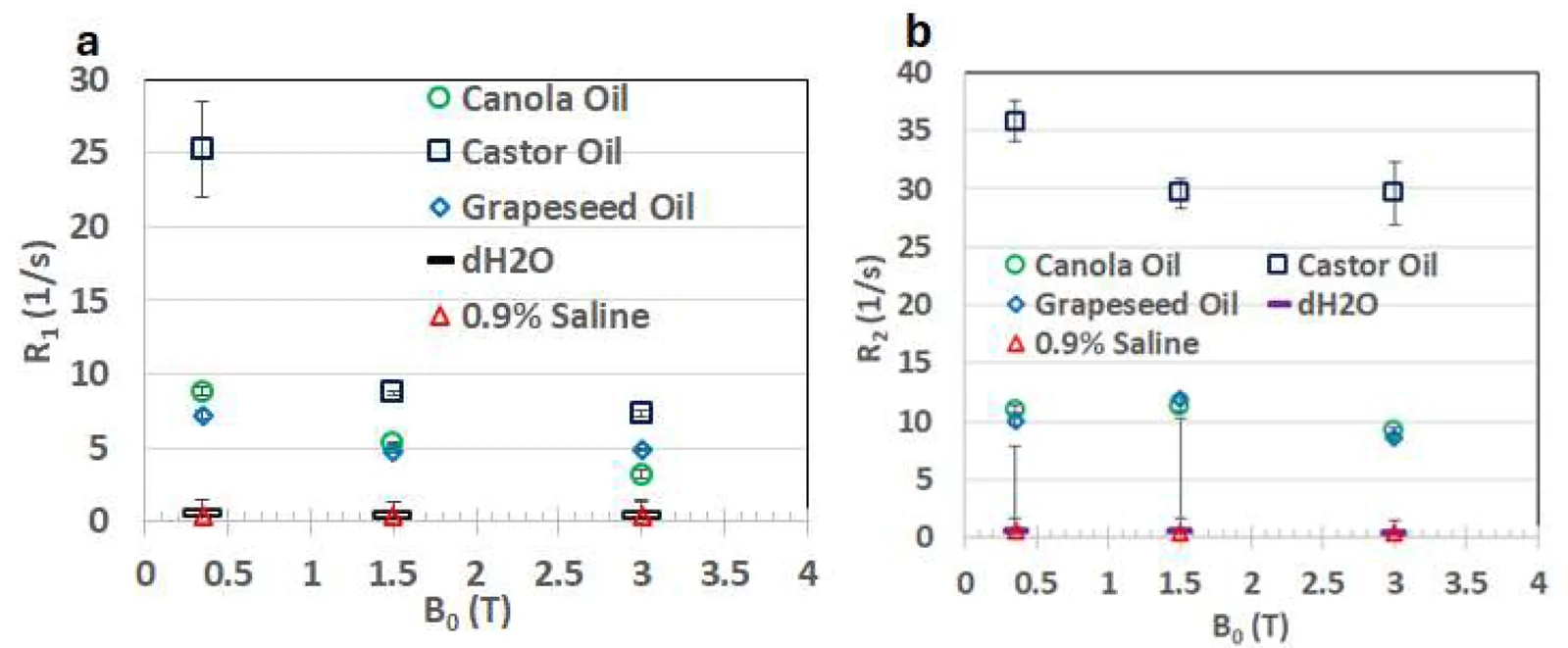
Relaxation rates for a) T_1_ and b) T_2._ for canola oil, castor, oil, grapeseed oil, deionized water, and 0.9% saline.(dH2O)

**TABLE 1:** T_1_ inversion recovery pulse sequence parameters

Parameter	0.35 T	1.5 T	3 T
TE	18 ms	15 ms	13 or 20 ms
TR	15 s	15 s	15 s
Flip angle	90°	90°	90°
TI’s	21–5000 ms 21,25,30,35,40,50,60,70, 80,90,100,120,140,160, 180,200,250,300,350, 400,450,500,600,750, 1000,1500,2000,3000, 4000,5000	30–5000 ms 30,35,40,50,60,70,80, 90,100,120,140,160, 180,200,250,300,350, 400,450,500,600,750, 1000,2000,3000,4000, 5000	21 or 34–5000 ms 21/34,40,50,60,70,80,90, 100,120,140,160,180, 200,250,300,350,400, 450,500,600,750,1000, 2000,3000,4000,5000
Bandwidth	2083 Hz/px	2050 Hz/px	3030 Hz/px
Resolution	3x3x5 mm^3^	3x3x5 mm^3^	3x3x5 mm^3^

**TABLE 2:** Nominal T_2_ map pulse sequence parameters

Parameter	0.35 T	1.5 T	3 T
TE	n*20 ms, n=1,32	n*20 ms, n=1,32	n*20 ms, n=1,32
TR	15 s	15 s	10 s
Flip angle	90°	90°	90°
Bandwidth	130 Hz/px	128 Hz/px	128 Hz/px
Resolution	3x3x5 mm^3^	3x3x5 mm^3^	3x3x5 mm^3^

**TABLE 3:** Miller's LB agar mixture relaxation times and fit uncertainties in ms measured at room temperature 20°C. Units: T_1_ and T_2_ (ms), r_1_ and r_2_ (w/%w-s), R_1,0_ and R_2,0_ (1/s), *a* (w/%w), *b* (S/m∙w/%w), σ_s,0_ (S/m).

Concentration (%w/w)		0.35 T (14.71 MHz)	1.5 T (63.89 MHz)	3 T (123.21 MHz)
0.5	T_1_	3036.76 ± 53.45	3055.48 ± 10.69	3271.60 ± 22.62
	T_2_	1112.92 ± 35.64	1155.31 ± 39.89	1207.02 ± 14.29
1	T_1_	2869.06 ± 84.47	3086.54 ± 14.47	3150.17 ± 27.86
	T_2_	428.51. ± 5.65	396.03 ± 5.14	376.29 ± 6.07
2	T_1_	2662.90 ± 23.25	2889.71 ± 36.00	3193.20 ± 28.56
	T_2_	361.56 ± 4.92	290.66 ± 3.69	283.51 ± 4.23
3	T_1_	2546.08 ± 31.48	2674.53 ± 11.16	2849.65 ± 23.05
	T_2_	185.56 ± 1.20	157.88 ± 1.26	157.92 ± 2.08
4	T_1_	2340.55 ± 19.45	2553.39 ± 12.89	2752.67 ± 21.40
	T_2_	162.13 ± 12.46	139.17 ± 0.93	149.16 ± 2.08
5	T_1_	2098.00 ± 41.01	2475.63 ± 8.83	2752.67 ± 18.81
	T_2_	123.92 ± 6.26	104.10 ± 0.57	102.63 ± 1.80
Relaxivities	r_1_	0.030± 0.00	0.02 ± 0.00	0.01 ± 0.00
	R_1,0_	0.31 ± 0.01	0.31 ± 0.00	0.30 ± 0.01
	r_2_	1.53 ± 0.13	1.77 ± 0.12	1.73 ± 0.16
	R_2,0_	0.31 ± 0.38	0.51 ± 0.37	0.59 ± 0.49
Permittivities				
Dielectric		Conductivity		DC Conductivity
ε_s,0_	7.98E1 ± 4.93E-1	σ_s,0_	5.10E-2 ± 3.93E-2	8.22E-2 ± 3.48E-2
*a*	−7.88E-1 ± 1.62E-1	*b*	3.87E-1 ± 1.30E-2	3.81E-1 ± 1.15E-2

**TABLE 4:** Gelatin relaxation times and fit uncertainties in ms measured at room temperature 20°C. Units: T_1_ and T_2_ (ms), r_1_ and r_2_ (w/%w-s), R_1,0_ and R_2,0_ (1/s), *a* (w/%w), *b* (S/m∙w/%w), σ_s,0_ (S/m).

Concentration (%w/w)		0.35 T (14.71 MHz)	1.5 T (63.89 MHz)	3 T (123.21 MHz)
0.5	T_1_	2353.39 ± 46.44	2578.61 ± 9.26	2910.22 ± 24.83
	T_2_	2013.33 ± 254.63	2562.23 ± 177.67	2203.17 ± 68.38
1	T_1_	2553.88 ± 322.93	2594.99 ± 10.73	2180.51 ± 128.70
	T_2_	2026.55 ± 95.43	2134.89 ± 122.63	2213.18 ± 71.89
2	T_1_	2191.25 ± 54.01	2502.77± 8.69	2701.10 ± 20.67
	T_2_	1639.22 ± 123.04	1400.78 ± 18.10	1343.08 ± 29.57
3	T_1_	1739.71 ± 40.90	2401.19 ± 8.41	2542.23 ± 27.17
	T_2_	1395.19 ± 92.56	1569.98 ± 193.79	1363.78 ± 31.45
4	T_1_	1977.73 ± 35.51	2292.92 ± 12.54	2499.97 ± 15.20
	T_2_	1166.15 ± 45.58	1063.30 ± 23.56	988.80 ± 12.49
5	T_1_	1821.11 ± 24.43	2159.51 ± 8.98	2395.55 ± 26.14
	T_2_	1049.05 ± 22.85	1017.94 ± 6.19	956.20 ± 16.03
Relaxivities	r_1_	0.03 ± 0.01	0.02 ± 0.00	0.01 ± 0.01
	R_1,0_	0.40 ± 0.04	0.37 ± 0.01	0.38 ± 0.03
	r_2_	0.11 ± 0.01	0.13 ± 0.02	0.14 ± 0.20
	R_2,0_	0.41 ± 0.02	0.35 ± 0.06	0.36 ± 0.06
Permittivities				
Static Dielectric		Static Conductivity		DC Conductivity
ε_s,0_	7.99E1 ± 8.57E-1	σ_s,0_	1.68E-3 ± 4.71E-3	5.11E-3 ± 5.11E-4
*a*	−2.28E-1 ± 2.82E-1	*b*	1.34E-2 ± 1.55E-3	7.82E-3 ± 1.69E-4

**TABLE 5:** PEG relaxation times and fit uncertainties in ms measured at room temperature 20°C. Units: T_1_ and T_2_ (ms), r_1_ and r_2_ (1/mM-s), R_1,0_ and R_2,0_ (1/s), *a* (1/mM), *b* (S/m∙mM), σ_s,0_ (S/m).

Concentration (mM)		0.35 T (14.71 MHz)	1.5 T (63.89 MHz)	3 T (123.21 MHz)
1	T_1_	2451.57 ± 15.39	2654.44 ± 10.64	2877.36 ± 38.25
	T_2_	1760.73 ± 97.93	2152.27 ± 98.11	2175.70 ± 85.22
2	T_1_	2461.77 ± 18.78	2619.44 ± 7.55	2692.26 ± 100.80
	T_2_	1385.41 ± 183.30	1646.89 ± 73.65	2297.23 ± 46.03
3	T_1_	2462.23 ± 20.87	2662.66 ± 8.20	2958.85 ± 48.90
	T_2_	1628.87 ± 158.45	2522.98 ± 144.89	2499.99 ± 75.04
4	T_1_	2472.98 ± 20.35	2690.55 ± 10.13	2759.24 ± 66.67
	T_2_	1595.33 ± 152.45	2665.09 ± 147.51	2327.21 ± 58.87
5	T_1_	2447.27 ± 19.81	2657.64 ± 9.46	2215.34 ± 111.17
	T_2_	1395.39 ± 160.76	1981.40 ± 79.54	2085.52 ± 31.06
10	T_1_	2457.10 ± 17.60	2610.58 ± 10.53	2741.32 ± 33.84
	T_2_	1520.81 ± 151.64	2218.64 ± 139.03	2086.51 ± 79.95
20	T_1_	2465.77 ± 30.45	2579.28 ± 6.79	2877.65 ± 14.25
	T_2_	1652.47 ± 79.40	1836.97 ± 75.37	2102.10 ± 44.60
Relaxivities	r_1_	0.00 ± 0.00	0.00 ± 0.00	0.00 ± 0.00
	R_1,0_	0.41 ± 0.00	0.37 ± 0.00	0.37 ± 0.02
	r_2_	0.00 ± 0.00	0.00 ± 0.01	0.00 ± 0.00
	R_2,0_	0.65 ± 0.03	0.46 ± 0.05	0.43 ± 0.02
Permittivities				
Static Dielectric		Static Conductivity		DC Conductivity
ε_s,0_	7.86E1 ± 3.03E-2	σ_s,0_	−2.99E-4 ± 1.55E-4	1.36E-4 ± 2.58E-5
*a*	−4.69E-3 ± 3.63E-3	*b*	4.07E-5 ± 1.86E-5	8.72E-6 ± 3.10E-6

**TABLE 6:** PVA relaxation times and fit uncertainties in ms measured at room temperature 20°C. Units: T_1_ and T_2_ (ms), r_1_ and r_2_ (w/%w-s), R_1,0_ and R_2,0_ (1/s), *a* (w/%w), *b* (S/m∙w/%w), σ_s,0_ (S/m).

Concentration (%w/w)		0.35 T (14.71 MHz)	1.5 T (63.89 MHz)	3 T (123.21 MHz)
0.5	T_1_	2346.60 ± 28.88	2568.33 ± 10.74	2829.28 ± 23.50
	T_2_	1462.93 ± 80.57	1546.75 ± 60.35	1355.41 ± 31.40
1	T_1_	2301.15 ± 25.93	2526.82 ± 11.59	2781.07 ± 24.18
	T_2_	1551.56 ± 100.09	1410.71 ± 53.60	1147.34 ± 9.17
2	T_1_	2240.15 ± 37.56	2457.38 ± 21.00	2716.44 ± 17.02
	T_2_	1433.32 ± 57.80	1082.51 ± 15.88	853.67 ± 5.73
3	T_1_	2076.42 ± 24.92	2384.55 ± 10.67	2488.19 ± 22.26
	T_2_	1522.31 ± 81.15	1214.93 ± 28.82	796.51 ± 7.77
4	T_1_	2051.60 ± 15.38	2242.25 ± 18.63	2569.04 ± 20.09
	T_2_	1680.19 ± 67.68	1359.11 ± 49.23	1058.64 ± 12.60
5	T_1_	1922.64 ± 9.75	2198.22 ± 7.29	2402.47 ± 18.77
	T_2_	1477.18 ± 61.05	1010.65 ± 23.13	625.23 ± 4.91
Relaxivities	r_1_	0.02 ± 0.00	0.02 ± 0.00	0.01 ± 0.00
	R_1,0_	0.41 ± 0.01	0.38 ± 0.00	0.35 ± 0.01
	r_2_	−0.01 ± 0.01	0.05 ± 0.03	0.14 ± 0.06
	R_2,0_	0.68 ± 0.03	0.67 ± 0.09	0.73 ± 0.17
Permittivities				
Static Dielectric		Static Conductivity		DC Conductivity
ε_s,0_	7.97E1 ± 5.87E-1	σ_s,0_	5.82E-4 ± 1.81E-3	3.10E-3 ± 1.07E-3
*a*	−5.17E-1 ± 1.93E-1	*b*	8.43E-3 ± 5.95E-4	6.91E-3 ± 3.51E-4

**TABLE 7:** PVP Relaxation times and fit uncertainties in ms measured at room temperature 20°C. Units: T_1_ and T_2_ (ms), r_1_ and r_2_ (w/%w-s), R_1,0_ and R_2,0_ (1/s), *a* (w/%w), *b* (S/m∙w/%w), σ_s,0_ (S/m).

Concentration (%w/w)		0.35 T (14.71 MHz)	1.5 T (63.89 MHz)	3 T (123.21 MHz)
10	T_1_	1867.45 ± 16.04	2077.23 ± 8.71	2146.40 ± 27.68
	T_2_	1435.95 ± 80.32	1359.17 ± 46.13	1429.87 ± 13.73
15	T_1_	1647.79 ± 13.54	1740.09 ± 15.93	1932.05 ± 13.56
	T_2_	1461.10 ± 44.90	1450.21 ± 82.07	1481.08 ± 28.67
20	T_1_	1478.96 ± 18.45	1556.21 ± 7.39	1677.32 ± 10.41
	T_2_	1347.76 ± 37.36	1321.26 ± 30.61	1294.94 ± 21.24
25	T_1_	1249.93 ± 9.85	1323.69 ± 5.22	1352.30 ± 12.51
	T_2_	1132.60± 32.00	1153.72 ± 19.52	1087.10 ± 13.45
30	T_1_	1014.23 ± 12.32	1055.45 ± 4.06	1196.52 ± 5.39
	T_2_	968.13 ± 16.15	943.74 ± 14.72	928.17 ± 9.68
Relaxivities	r_1_	0.02 ± 0.00	0.02 ± 0.00	0.02 ± 0.00
	R_1,0_	0.28 ± 0.06	0.24 ± 0.06	0.25 ± 0.04
	r_2_	0.02 ± 0.00	0.02 ± 0.01	0.02 ± 0.00
	R_2,0_	0.46 ± 0.09	0.49 ± 0.11	0.44 ± 0.09
Permittivities				
Static Dielectric		Static Conductivity		DC Conductivity
ε_s,0_	8.49E1 ± 3.91E0	σ_s,0_	2.29E-2 ± 2.00E-3	1.71E-2 ± 1.90E-3
*a*	−9.62E-1 ± 1.84E-1	*b*	1.15E-3 ± 9.41E-5	1.76E-4 ± 8.94E-5

**TABLE 8:** Sodium alginate relaxation times and fit uncertainties in ms measured at room temperature 20°C. Units: T_1_ and T_2_ (ms), r_1_ and r_2_ (w/%w-s), R_1,0_ and R_2,0_ (1/s), *a* (w/%w), *b* (S/m∙w/%w), σ_s,0_ (S/m).

Concentration (%w/w)		0.35 T (14.71 MHz)	1.5 T (63.89 MHz)	3 T (123.21 MHz)
0.5	T_1_	2338.54 ± 12.69	2568.82 ± 7.13	2678.16 ± 65.82
	T_2_	1415.61 ± 71.43	1628.03 ± 53.96	1496.10 ± 25.88
1	T_1_	2260.97 ± 24.84	2485.53 ± 6.99	2732.23 ± 13.82
	T_2_	1197.80 ± 22.65	1127.44 ± 16.03	1144.77 ± 14.94
2	T_1_	2051.83 ± 14.36	2414.58 ± 5.18	2626.46 ± 47.17
	T_2_	840.36 ± 34.24	809.01 ± 6.54	782.93 ± 6.18
3	T_1_	1953.39 ± 7.00	2284.79 ± 7.91	2442.97 ± 51.60
	T_2_	681.28 ± 15.44	609.76 ± 4.02	569.29 ± 4.77
4	T_1_	1876.86 ± 12.26	2245.70 ± 3.14	2363.98 ± 71.47
	T_2_	531.04 ± 8.52	485.35 ± 3.81	460.28 ± 3.76
5	T_1_	1818.54 ± 9.38	2167.71 ± 7.55	2387.52 ± 35.11
	T_2_	585.09 ± 11.80	509.82 ± 3.38	503.63 ± 2.92
Relaxivities	r_1_	0.03 ± 0.00	0.02 ± 0.00	0.01 ± 0.00
	R_1,0_	0.42 ± 0.01	0.39 ± 0.00	0.36 ± 0.01
	r_2_	0.26± 0.04	0.32 ± 0.04	0.34 ± 0.05
	R_2,0_	0.63 ± 0.17	0.56 ± 0.13	0.58 ± 0.17
Permittivities				
Static Dielectric		Static Conductivity		DC Conductivity
ε_s,0_	7.93E1 ± 1.08E0	σ_s,0_	4.09E-2 ± 9.25E-3	5.84E-2 ± 2.20E-2
*a*	−4.53E-1 ± 3.55E-1	*b*	1.07E-1 ± 3.05E-3	1.20E-1 ± 7.25E-3

**TABLE 9:** Sodium polyacrylate relaxation times and fit uncertainties in ms measured at room temperature 20°C. Units: T_1_ and T_2_ (ms), r_1_ and r_2_ (w/%w-s), R_1,0_ and R_2,0_ (1/s), *a* (w/%w), *b* (S/m∙w/%w), σ_s,0_ (S/m).

Concentration (%w/w)		0.35 T (14.71 MHz)	1.5 T (63.89 MHz)	3 T (123.21 MHz)
0.25	T_1_	1509.67 ± 10.58	1663.39 ± 3.10	1945.18 ± 23.01
	T_2_	1160.79 ± 251.24	1068.51 ± 13.34	1158.53 ± 11.81
1	T_1_	1400.09 ± 18.02	1515.42 ± 2.92	1808.76 ± 8.20
	T_2_	1099.77 ± 52.17	885.06 ± 11.17	832.69 ± 9.98
3	T_1_	1328.95 ± 6.15	1446.53 ± 10.64	1620.51 ± 27.36
	T_2_	1052.61 ± 34.79	919.04 ± 11.52	765.50 ± 5.35
4	T_1_	1353.54 ± 10.31	1455.70 ± 4.95	1640.39 ± 30.76
	T_2_	1027.08 ± 53.71	868.12 ± 7.46	793.70 ± 8.05
5	T_1_	1364.60 ± 9.65	1447.16 ± 6.05	1670.88 ± 13.31
	T_2_	1022.09 ± 31.39	869.33 ± 7.86	827.81 ± 10.63
Relaxivities	r_1_	0.02 ± 0.01	0.02 ± 0.00	0.02 ± 0.00
	R_1,0_	0.66 ± 0.03	0.61 ± 0.01	0.53 ± 0.01
	r_2_	0.04 ± 0.03	0.11 ± 0.01	0.18 ± 0.01
	R_2,0_	0.93 ± 0.09	1.09 ± 0.04	1.19 ± 0.03
Permittivities				
Static Dielectric		Static Conductivity		DC Conductivity
ε_s,0_	7.57E1 ± 1.98E0	σ_s,0_	−1.76E-3 ± 3.20E-2	1.45E-2 ± 1.72E-2
*a*	−3.27E-1 ± 7.53E-1	*b*	2.18E-1 ± 1.22E-2	1.73E-1 ± 6.55E-3

**TABLE 10:** Xanthan gum relaxation times and fit uncertainties in ms measured at room temperature (20°C). Units: T_1_ and T_2_ (ms), r_1_ and r_2_ (w/%w-s), R_1,0_ and R_2,0_ (1/s), *a* (w/%w), *b* (S/m∙w/%w), σ_s,0_ (S/m).

Concentration (%w/w)		0.35 T (14.71 MHz)	1.5 T (63.89 MHz)	3 T (123.21 MHz)
0.5	T_1_	1875.67 ± 12.51	2111.68 ± 6.05	2339.91 ± 11.19
	T_2_	653.14 ± 9.93	573.95 ± 3.24	556.93 ± 15.94
1	T_1_	1845.74 ± 7.85	2121.07 ± 6.70	2314.78 ± 10.43
	T_2_	596.84 ± 9.69	556.57 ± 5.16	564.19 ± 24.21
2	T_1_	1780.70 ± 12.05	2125.67 ± 5.22	2349.65 ± 15.65
	T_2_	297.89 ± 3.26	285.74 ± 3.50	344.01 ± 2.15
3	T_1_	1545.06 ± 19.52	1866.93 ± 2.67	2130.01 ± 11.89
	T_2_	166.79 ± 3.92	167.83 ± 1.41	177.94 ± 1.19
4	T_1_	1279.84 ± 8.01	1601.64 ± 3.14	1924.59 ± 14.12
	T_2_	137.05 ± 2.49	122.09 ± 1.82	153.27 ± 4.91
5	T_1_	1166.64 ± 11.42	1477.87 ± 4.01	1754.36 ± 8.94
	T_2_	115.39 ± 0.39	112.98 ± 1.08	127.75 ± 0.60
Relaxivities	r_1_	0.08 ± 0.01	0.05 ± 0.01	0.03 ± 0.01
	R_1,0_	0.46 ± 0.03	0.42 ± 0.03	0.39 ± 0.02
	r_2_	1.71 ± 0.11	1.77 ± 0.14	1.47 ± 0.14
	R_2,0_	0.34 ± 0.34	0.43 ± 0.44	0.63 ± 0.42
Permittivities				
Static Dielectric		Static Conductivity		DC Conductivity
ε_s,0_	8.13E1 ± 4.00E0	σ_s,0_	1.45E-2 ± 1.38E-2	1.96E-2 ± 5.49E-3
*a*	−1.57E0 ± 1.32E0	*b*	5.56E-2 ± 4.56E-3	4.82E-2 ± 1.81E-3

**TABLE 11:** CuSO_4_ relaxation times and fit uncertainties in ms measured at room temperature 20°C

Concentration (mM)		0.35 T (14.71 MHz)	1.5 T (63.89 MHz)	3 T (123.21 MHz)
0.5	T_1_	1058.52 ± 8.16	1240.05 ± 1.63	1361.51 ± 12.29
	T_2_	887.42 ± 45.11	1093.77 ± 10.75	1183.18 ± 9.82
1	T_1_	670.29 ± 10.88	838.55 ± 8.17	1035.99 ± 6.35
	T_2_	633.60 ± 27.37	804.33 ± 27.60	902.47 ± 49.37
2	T_1_	376.12 ± 5.83	536.44 ± 0.59	542.48 ± 10.70
	T_2_	365.77 ± 3.62	448.03 ± 12.47	525.49 ± 18.77
5	T_1_	174.59 ± 2.14	252.29 ± 3.05	250.99 ± 6.02
	T_2_	164.41 ± 1.53	219.51 ± 1.80	245.08 ± 2.60
10	T_1_	82.59 ± 3.34	125.04 ± 0.76	160.10 ± 3.34
	T_2_	82.15 ± 0.45	110.21 ± 0.50	121.56 ± 2.20
15	T_1_	59.65 ± 1.20	86.50 ± 0.25	92.83 ± 0.38
	T_2_	54.94 ± 0.45	74.87 ± 0.42	80.59 ± 0.33
20	T_1_	46.91 ± 2.53	64.48 ± 0.15	80.32 ± 1.92
	T_2_	38.61 ± 0.88	55.58 ± 0.39	61.35 ± 0.53
Relaxivities	r_1_	1.06 ± 0.03	0.75 ± 0.01	0.62 ± 0.03
	R_1,0_	0.64 ± 027	0.35 ± 0.03	0.52 ± 0.31
	r_2_	1.25 ± 0.03	0.87 ± 0.01	0.80 ± 0.01
	R_2,0_	0.14 ± 0.31	0.38 ± 0.08	0.28 ± 0.06
Permittivities				
Static Dielectric		Static Conductivity		DC Conductivity
ε_s,0_	7.88E1 ± 6.67E-1	σ_s,0_	1.95E-2 ± 7.86E-3	1.13E-2 ± 3.15E-3
*a*	1.24E-1 ± 5.67E-2	*b*	9.62E-3 ± 6.69E-4	1.09E-2 ± 3.03E-4

**TABLE 12:** Mn(NO_3_)_2_ relaxation times and fit uncertainties in ms measured at room temperature (20°C)

Concentration (mM)		0.35 T (14.71 MHz)	1.5 T (63.89 MHz)	3 T (123.21 MHz)
0.01	T_1_	2043.11 ± 37.10	2202.60 ± 5.46	2469.91 ± 15.16
	T_2_	1009.49 ± 37.12	815.98 ± 4.41	672.59 ± 5.36
0.02	T_1_	1771.33 ± 6.66	2015.92 ± 5.21	2276.35 ± 34.71
	T_2_	966.72 ± 16.81	637.61 ± 3.75	505.15 ± 4.04
0.05	T_1_	844.51 ± 7.99	1061.77 ± 1.57	1201.41 ± 17.79
	T_2_	311.15 ± 5.51	152.56 ± 2.34	112.75 ± 5.17
0.1	T_1_	574.24 ± 3.77	746.85 ± 4.62	815.71 ± 3.41
	T_2_	198.02 ± 2.68	109.99 ± 0.67	77.19 ± 0.89
0.2	T_1_	369.90 ± 4.05	464.32 ± 0.91	548.32 ± 12.29
	T_2_	123.71 ± 0.85	67.06 ± 0.41	45.92 ± 0.69
0.5	T_1_	171.62 ± 1.68	226.82 ± 0.81	242.84 ± 1.50
	T_2_	49.34 ± 0.74	24.25 ± 0.82	17.09 ± 0.67
1	T_1_	87.31 ± 1.40	118.73 ± 1.45	224.37 ± 19.08
	T_2_	24.53 ± 1.09	11.04 ± 0.87	8.37 ± 0.67
2	T_1_	41.82 ± 1.32	55.05 ± 4.87	171.85 ± 36.43
	T_2_	13.32 ± 1.22	6.90 ± 1.09	*
3	T_1_	10.94 ± 1.34	46.81 ± 3.35	*
	T_2_	7.42 ± 0.42	4.12 ± 0.46	*
Relaxivities	r_1_	8.60 ± 0.71	8.62 ± 0.34	2.86 ± 0.55
	R_1,0_	2.10 ± 1.30	0.26 ± 0.28	1.09 ± 0.45
	r_2_	37.46 ± 0.50	73.80 ± 2.55	118.35 ± 1.91
	R_2,0_	1.08 ± 0.41	2.36 ± 2.08	0.36 ± 0.82
Permittivities				
Static Dielectric		Static Conductivity		DC Conductivity
ε_s,0_	7.83E1 ± 1.04E0	σ_s,0_	4.19E-3 ± 2.07E-3	1.19E-3 ± 7.83E-4
*a*	5.10E-2 ± 4.65E-1	*b*	1.55E-2 ± 9.21E-4	1.86E-2 ± 3.49E-4

**TABLE 13:** NiCl_2_ relaxation times and fit uncertainties in ms measured at room temperature (20°C)

Concentration (mM)		0.35 T (14.71 MHz)	1.5 T (63.89 MHz)	3 T (123.21 MHz)
0.5	T_1_	1341.56 ± 31.87	1293.22 ± 2.89	1324.84 ± 12.29
	T_2_	1084.03 ± 14.17	1058.52 ± 12.69	969.98 ± 11.06
1	T_1_	1052.18 ± 8.05	1031.44 ± 2.57	1023.46 ± 4.22
	T_2_	927.20 ± 9.69	860.28 ± 4.24	792.25 ± 3.84
2	T_1_	543.86 ± 3.47	513.58 ± 3.04	502.96 ± 19.60
	T_2_	504.28 ± 5.25	447.17 ± 12.47	381.18 ± 0.49
5	T_1_	314.77 ± 3.14	294.12 ± 3.24	288.09 ± 3.78
	T_2_	307.58 ± 4.89	262.23 ± 1.41	217.20 ± 1.12
10	T_1_	168.16 ± 12.34	155.57 ± 0.58	148.49 ± 3.78
	T_2_	167.11 ± 0.67	137.91 ± 0.59	115.52 ± 0.55
15	T_1_	113.91 ± 2.61	107.37 ± 0.13	120.17 ± 4.04
	T_2_	112.24 ± 0.60	92.97 ± 0.30	75.34 ± 0.63
20	T_1_	101.07 ± 4.40	82.82 ± 0.95	76.51 ± 4.04
	T_2_	85.49 ± 0.46	69.68 ± 0.29	58.53 ± 0.41
Relaxivities	r_1_	0.51 ± 0.03	0.60 ± 0.01	0.61 ± 0.03
	R_1,0_	0.45 ± 0.31	0.26 ± 0.15	0.20 ± 0.35
	r_2_	0.56 ± 0.00	0.69 ± 0.01	0.83 ± 0.01
	R_2,0_	0.54 ± 0.04	0.44 ± 0.06	0.49 ± 0.09
Permittivities				
Static Dielectric		Static Conductivity		DC Conductivity
ε_s,0_	7.88E1 ± 1.32E-1	σ_s,0_	3.09E-3 ± 1.05E-2	5.02E-3 ± 2.67E-3
*a*	2.61E-2 ± 1.36E-2	*b*	1.32E-2 ± 1.08E-3	1.77E-2 ± 2.57E-4

**TABLE 14:** Liquid relaxation times and fit uncertainties in ms measured at room temperature (20°C)

Sample		0.35 T (14.71 MHz)	1.5 T (63.89 MHz)	3 T (123.21 MHz)
Canola oil	T_1_	126.87 ± 4.46	182.24 ± 2.98	214.11 ± 18.90
	T_2_	97.51 ± 1.87	106.09 ± 2.21	127.72 ± 1.29
Castor oil	T_1_	44.10 ± 8.05	123.56 ± 2.70	143.44 ± 16.46
	T_2_	24.75 ± 0.75	33.84 ± 0.70	40.15 ± 0.79
Grapeseed oil	T_1_	133.67 ± 9.58	215.21 ± 4.59	258.88 ± 1.63
	T_2_	85.86 ± 3.00	101.97 ± 1.83	116.37 ± 6.76
dH_2_O	T_1_	2184.01 ± 46.67	2563.73 ± 6.22	2522..47 ± 40.20
	T_2_	2051.72 ± 12.84	1755.22 ± 13.42	2422.19 ± 16.67
0.9% Saline	T_1_	2466.16 ± 17.57	2622.79 ± 18.14	2791.69 ± 12.57
	T_2_	1786.26 ± 130.187	2458.1 ± 112.11	2724.71 ± 259.18

**TABLE 15:** Permittivities and conductivities measured at room temperature (20°C)

Sample	ε_s_	σ_s_ (S/m)
Canola oil	4.96E0 ± 1.69E0	6.87E-4 ± 4.30E-5
Castor oil	3.16E0 ± 1.27E-2	1.98E-3 ± 1.03E-4
Grapeseed oil	3.34E0 ± 1.5E-3	5.00E-3 ± 5.44E-4
dH_2_O	7.96E1 ± 1.06E-3	1.54E-4 ± 4.38E-5
0.9% Saline	7.74E1 ± 5.80E-3	1.58E0 ± 6.50E-4

## Data Availability

Data may be provided upon request.

## HIGHLIGHTS:

Relaxation times and complex permittivities for three types of materials (hydrogels, paramagnetic salt solutions, and oils) commonly used in MRI phantoms are measured at three magnetic field strengths (0.35, 1.5, and 3 T), interpolated for 0.55 T, and extrapolated for 7 T. Assumptions that T_1_ rises, or T_1_ relaxivity (r_1_) decreases, with increasing field strength was not true for some of our samples under test.
