# Supplementary material for: T1, T2, and complex permittivities of hydrogels, paramagnetic salt solutions, and oils at 0.35, 1.5, and 3 Tesla
Source: ArXiv. 2026 Aug 11:arXiv:2608.11117v1. Preprint. [Version 1] (PMC13640857)
Supplement: Supplementary file 1 [file NIHPP2608.11117v1-supplement-1.pdf]

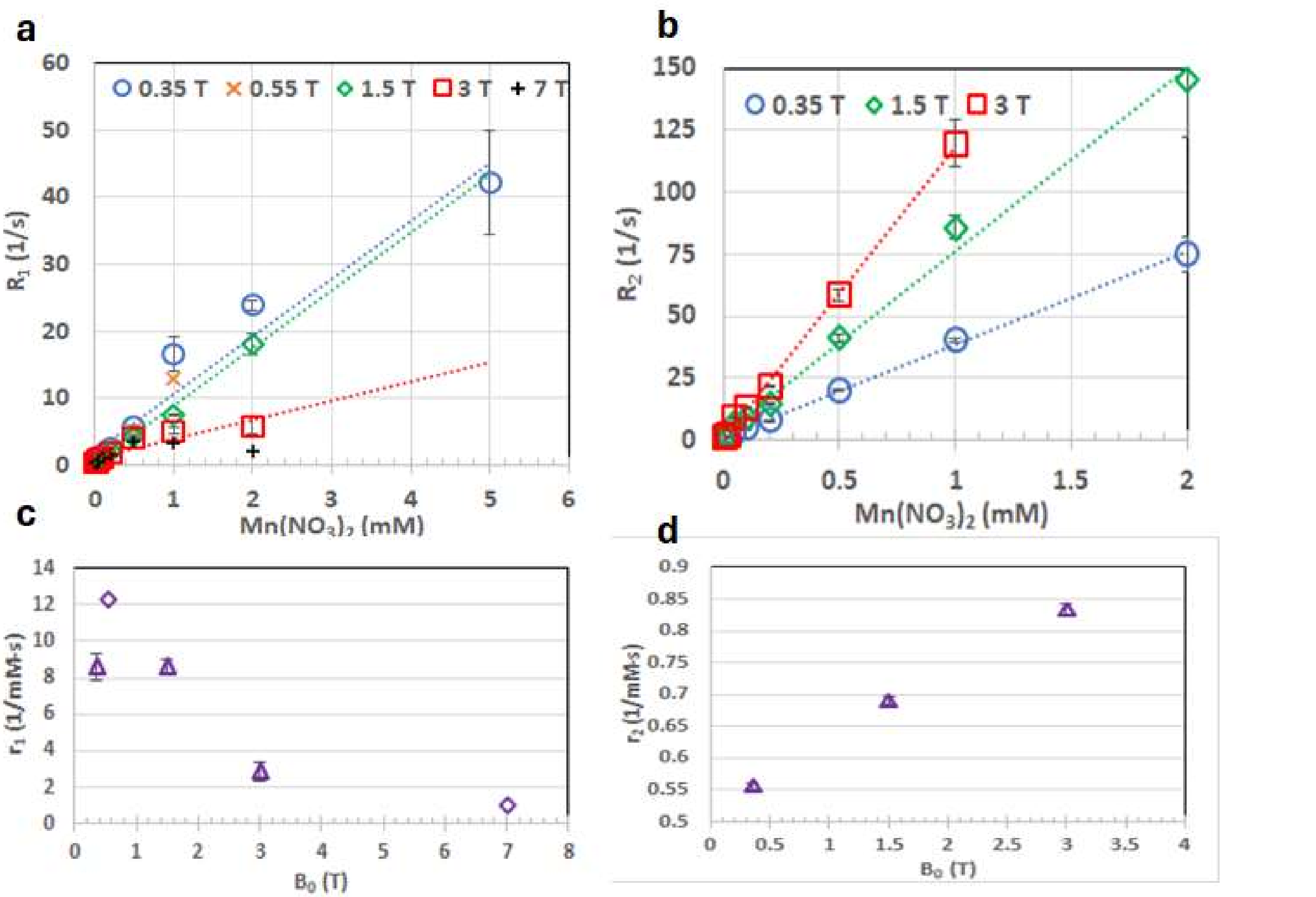

FIGURE S19 Relaxation rates for different concentrations of $Mn(NO_3)_2$: a) R_1 and b) R_2, and $Mn(NO_3)_2$ relaxivities vs. field strength for c) r_1 and d) r_2. R_1 (×, +) and r_1 (◊) interpolations/extrapolations are included for 0.55 T and 7 T. Signal dephasing was dependent on field strength and limited the range of paramagnetic salt concentrations.
